# Acteoside attenuates RSV-induced lung injury by suppressing necroptosis and regulating metabolism

**DOI:** 10.3389/fphar.2022.870928

**Published:** 2022-08-19

**Authors:** Xiaoying Ling, Jie Zhou, Tianzi Jin, Weichen Xu, Xun Sun, Weifeng Li, Yali Ding, Miaomiao Liang, Chenbi Zhu, Peipei Zhao, Chanchan Hu, Bin Yuan, Tong Xie, Jialei Tao

**Affiliations:** ^1^ Department of Pediatrics, Affiliated Hospital of Nanjing University of Chinese Medicine, Nanjing, China; ^2^ Jiangsu Key Laboratory of Pediatric Respiratory Disease, Institute of Pediatrics, Medical Metabolomics Center, Nanjing University of Chinese Medicine, Nanjing, China

**Keywords:** acteoside, respiratory syncytial virus, necroptosis, mitochondria, metabolomics

## Abstract

**Background:** Necroptosis and inflammation are closely related to the pathogenesis of respiratory syncytial virus (RSV). Acteoside (AC), a natural phenylpropanoid glycoside from Kuding Tea, has significant anti-RSV effect. However, the roles of AC on RSV-induced lung necroptosis and inflammation are yet to be elucidated.

**Methods:** The effects of AC were investigated in BALB/c mice and A549 cells. Lung histopathology was observed through H&E staining. The viral titer was assessed via plaque assay. The RSV-F expression was determined by RT-qPCR and immunohistochemistry assay. The levels of cytokines were detected by ELISA and RT-qPCR. The necroptosis rate and mitochondrial membrane potential were evaluated via flow cytometry. The expressions of HMGB1/NF-κB and RIP1/RIP3/MLKL/PGAM5/DRP1 were detected by western blot. Additionally, untargeted metabolomics was conducted to investigate the metabolic profiles and related metabolic pathways via Gas Chromatography-Mass Spectrometry.

**Results:** The results showed that compared with the RSV-infected group, AC treatment significantly attenuated lung pathological damage, virus replication, and cytokines levels. AC also alleviated RSV-induced necroptosis and mitochondrial dysfunction *in vitro and in vivo*. Moreover, AC treatment down-regulated the expression of HMGB1, p-Iκbα/Iκbα, p-p65/p65, RIP1, RIP3, MLKL, PGAM5, and DRP1. Furthermore, metabolomic analyses suggested that the perturbations in major metabolites of AC therapy were related to variations in amino acid and energy metabolism.

**Conclusion:** Our findings validated the beneficial effects of AC in suppressing necroptosis and regulating metabolism, suggesting AC may be a new drug candidate for RSV infection.

## Introduction

Respiratory syncytial virus (RSV), an enveloped negative-sense RNA virus, is the primary cause of acute respiratory infections in children, such as bronchiolitis and pneumonia (Bianchini et al., 2020). Moreover, severe RSV infection in infants facilitates the progression of recurrent wheezing and asthma (Jartti and Gern, 2017). Ribavirin and palivizumab are currently approved to treat and prevent RSV infection. However, ribavirin use is restricted to the cases of severe infection in immunocompromised patients due to its potential toxicity. Palivizumab can only be administered as a preventive treatment to patients with high risk of RSV-related complications, and its wider application is limited by the high cost (Domachowske et al., 2021). There is no clinically approved vaccine for RSV (Chatterjee et al., 2021). Owing to the lack of effective treatments against RSV, interest in natural compounds is rising among researchers.

**FIGURE 1:**
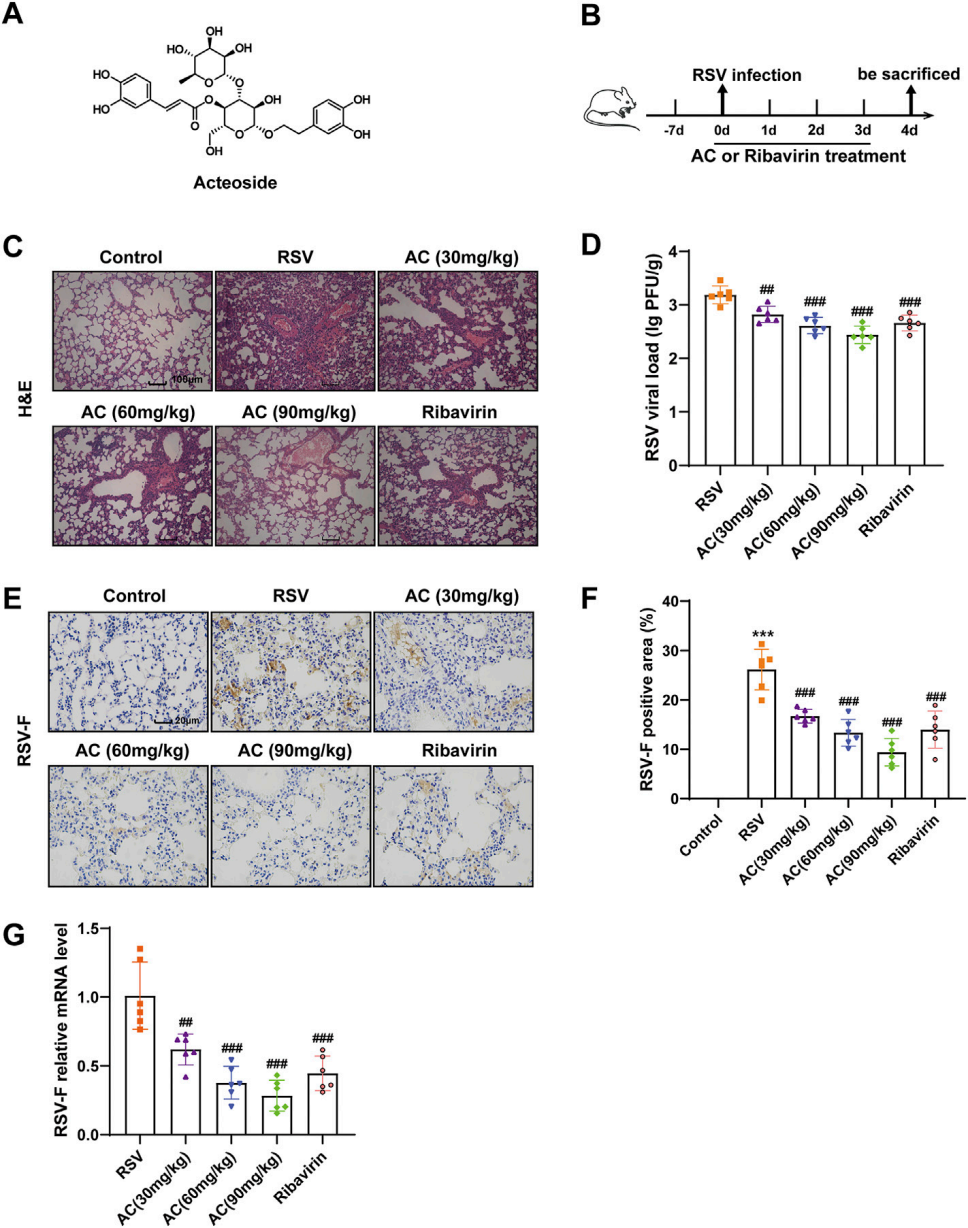
AC mitigated lung injury and inhibited virus replication in RSV-infected mice. **(A)** Chemical structure of AC. **(B)** Design of this study. **(C)** Typical H&E-stained lung tissues. Scale bar, 100 μm. **(D)** RSV viral load in lung tissues measured via plaque assay. **(E,F)** RSV-F protein expression assessed via immunohistochemistry. Scale bar, 20 μm. **(G)** RSV-F mRNA level identified via qPCR. Data are described as the mean ± SD (n = 6). ***p < 0.001, compared to the controls. ^##^ *p* < 0.01, and ^###^ *p* < 0.001 vs. the RSV group.

Acteoside (AC, Figure 1A), also known as verbascoside, is a natural phenylpropanoid glycoside acquired from *Ligustrum robustum* (Roxb.) Blume (Oleaceae) leaves. Ligustrum robustum, conventionally known as “Kuding Tea”, has been taken as a functional tea in China for approximately 2000 years. AC shows multiple pharmacological activities including anti-inflammatory, antioxidant, and antiviral functions (Song et al., 2016; Zheng et al., 2021). A recent study found that AC therapy ameliorated lipopolysaccharide-induced acute lung injury by suppressing inflammatory events mediated by NF-κB activation (Jing et al., 2015). Furthermore, AC therapy alleviated oxidative stress and suppressed mitochondrial damages in mice with experimental autoimmune encephalomyelitis (Li et al., 2020). In addition, the efficacy of AC in inhibiting RSV replication has been verified (Chathuranga et al., 2019). However, the role of AC on RSV-induced lung injury and its potential underlying mechanism merit further investigation.

The pathological mechanism of RSV, in which cell death plays a vital role, is complex. Necroptosis is a novel type of caspase-independent apoptosis that has been identified in recent years (Santos et al., 2021). The classical necroptosis pathway is initiated by receptor interacting protein kinase 1 (RIP1) and RIP3. Subsequently, RIP3 recruits and activates mixed-lineage kinase domain-like (MLKL), causing membranous rupture and necroptosis (Weinlich et al., 2017). Moreover, necrosomes composed of RIP1/3 can also stimulate mitochondrial phosphatase phosphoglycerate mutase family member 5 (PGAM5), subsequently recruiting and activating mitochondrial fission factor dynamin-related protein 1 (DRP1) (Wang et al., 2012). The stimulation of DRP1 induces mitochondrial division and reactive oxygen species (ROS) production, promoting necrotic cell death. After cellular rupture and necrosis, some endocellular damage-associated molecular patterns (DAMP) such as high-mobility group box 1 (HMGB1) are released (Zhang et al., 2020). HMGB1 further activates the NF-κB pathway, triggering inflammatory events and causing secondary injury. RSV has been shown to induce necroptosis, and inhibition of necroptosis can lower the viral load and attenuate inflammatory injury (Simpson et al., 2020), suggesting that inhibiting necroptosis is an appropriate target for therapeutic interventions.

Herein, our team first investigated whether AC played protective roles in RSV-induced lung inflammation and necroptosis both *in vivo* and *in vitro*. Meanwhile, the expressions of HMGB1/NF-κB and RIP1/RIP3/MLKL/PGAM5/DRP1 were evaluated to elucidate the potential molecular mechanisms involved. In addition, untargeted metabolomics was conducted to investigate the metabolic profiles and related metabolic pathways, thus providing insights regarding the metabolic mechanism of AC treatment.

## Materials and methods

### Materials

Acteoside (purity ≥98%, HPLC) was provided by JinYibai Biological Technology (Nanjing, China). Ribavirin (purity ≥98%, HPLC) was provided by Yuanye Bio-Technology Company (Shanghai, China). 1,2-13C-myristic acid, N, O-bis (trimethylsilyl) trifluoroacetamide (BSTFA), pyridine and methoxylamine salt were supplied by Sigma-Aldrich (Saint Louis, USA). Antibodies against Iκbα, NF-κB p65, E-Cadherin, MLKL, DRP1 and HMGB1 were bought from Proteintech (Wuhan, China). Antibodies against GAPDH, p-Iκbα, p-NF-κB p65, p-RIP1 were purchased from Affinity Biosciences (Changzhou, China). Antibodies against p-MLKL, p-RIP3 were supplied by Abcam (Cambridge, United Kingdom). Antibodies against RIP1 was bought from Cell Signaling Technology (Danvers, USA). Antibodies against RIP3, PGAM5 were supplied by Abmart (Shanghai, China). Antibody against RSV-F was bought from Santa Cruz Biotechnology (California, USA).

### Animals and treatment

Female BALB/c mice aged 6–8 weeks were supplied by Skbex Biotechnology Co., Ltd. (Henan, China) [No. SCXK (Yu) 2020-0005]. Our experiment was completed as per the National Institutes of Health Guidelines for Laboratory Animals and approved by the Animal Ethical Board of Nanjing University of Chinese Medicine (No: 201805A019). Following adaptive feeding for 7 days, animals were randomly assigned to six groups (n = 8 per group): the control group, RSV group, AC administration groups at 30/60/90 mg/kg/day, and the positive control group with ribavirin at 46 mg/kg/day. Regarding RSV infection, animals were treated with 80 μL RSV A2 strain suspension (10^7^ PFU/ml) via intranasal drop infection under 5% isoflurane anesthesia. The controls were treated with sham infection using carrier fluid. After RSV infection for 12 h, mice in the treatment groups were exposed to ribavirin oropharyngeally or AC intra-peritoneally for 4 days consistently. The RSV-infected group and control group instead received normal saline. All animals were sacrificed on the fourth day.

### Histologic analysis

Paraffin-embedded lung tissues were sliced into 4-μm sections. After deparaffinization, the slices were stained with hematoxylin and eosin (H&E).

### Plaque assay

Viral titer of the lung tissue was assessed via plaque assay (Xu et al., 2015). Briefly, lung tissues were homogenized and centrifuged. Then, the supernatant was added to HEp-2 cells in 6-well plates. After incubation for 2 h, the medium was substituted with carboxymethyl cellulose sodium medium. The cells were further cultured for 4–5 days to evaluate the cytopathic effect. After fixation and staining, the number of plaques was counted.

### Immunohistochemistry assay

IHC staining was performed in accordance with the steps of deparaffinization, rehydration, antigen retrieval, and blocking. Subsequently, specimens were cultured with the antibody against RSV fusion (RSV-F) (1:200). HRP-conjugated secondary antibody was used to label the first antibody. Then, samples were cleaned in PBS, counterstained with hematoxylin, visualized using an optical microscope (Carl Zeiss, Germany), and analyzed using ImageJ program.

### Transmission electron microscopy

The lung sections were fixed with 2.5% glutaric dialdehyde and post-fixed with 4% OsO₄. Subsequently, the sections were dehydrated in a gradient series of alcohol concentrations, subjected to embedment in Epon 812, and polymerized. The ultra-thin slices were stained and observed via a TEM apparatus (Hitachi, Japan).

### Quantitative real-time polymerase chain reaction

The total lung tissue RNA was extracted using the Fast Pure Cell/Tissue Total RNA Isolation Kit (Vazyme Biotech co., ltd., China) and converted to cDNA via reverse transcription. SYBR green on a Step One Sequence Detection System was used to perform the RT-qPCR assay. The 2^−ΔΔCT^ formula was applied to compute the comparative abundance of genes, with β-actin as an internal control. Supplementary Table S1 detailed the primer sequences.

### Cell culture and treatment

The human adenocarcinoma alveolar epithelial cell line A549 and RSV were kindly offered by Jiangsu Key Laboratory of Pediatric Respiratory Disease. The cells were cultured in Dulbecco’s modified Eagle’s medium (DMEM, Basal Media, China) added with 10% fetal bovine serum (FBS, Life-iLab, China), 100 μg/ml streptomycin and 100 U/mL penicillin (Basal Media, China) under 37°C with a 5% CO2 environment. In some experiments, cells were pretreated for 2 h with AC, and then inoculated with RSV (multiplicity of infection [MOI] of 1).

### Flow cytometry for cell death

A549 cells were pretreated with AC for 2 h and then exposed to RSV for another 48 h. Then, the cells were harvested, washed with phosphate buffered saline (PBS, Servicebio, China), stained with annexin V-fluorescein isothiocyante (FITC)/propidium iodide (PI) kit (Vazyme Biotech co., ltd., China), and detected via flow cytometry. Annexin V (+) and PI(+) cells in the upper right quadrant were considered as necroptosis (Zhang H. et al., 2019).

### Determination of the mitochondrial membrane potential

The MMP was identified via JC-1 probe (Beyotime Biological Technology, China). A549 cells were pretreated with AC for 2 h and then exposed to RSV for another 24 h. After that, the cells were cleaned in PBS and incubated with JC-1 work solution (10 μM) under 37°C without light for 20 min. The cellular staining was identified via flow cytometry.

### Enzyme-linked immunosorbent assay

Tumor Necrosis Factor-α (TNF-α), Interleukin-1β (IL-1β), Interleukin-6 (IL-6), Lactate Dehydrogenase (LDH), and HMGB1 contents in bronchoalveolar lavage fluid (BALF) and cell supernatant were determined as per the instructions of the ELISA kits (Nanjing JinTing biotechnology Co., Ltd., China).

### Western blot assay

The lung and cell proteins were produced using RIPA buffering solution with protease and phosphatase inhibitors and then identified via the BCA assay kit (Thermo Fisher Scientific, USA). The same quantity of proteins was isolated *via* SDS-PAGE, and moved onto polyvinylidene difluoride membranes (Millipore, USA). Then, they were sealed and incubated overnight at 4°C with appropriate primary antibodies. Afterwards, the membranes were cultured with secondary antibodies and visualized via ECL UItra (New Cell and Molecular Biotech, China).

### Immunofluorescence assay

For IF staining, lung sections were blocked in 1% BSA and cultured with antibodies against p-p65, E-Cadherin, and p-MLKL overnight at 4°C. Afterwards, the slices were cultured with FITC-conjugated secondary antibody for 60 min under ambient temperature. Following staining with DAPI, all slides were observed by fluorescent microscopy (Carl Zeiss, Germany).

A549 cells were pretreated with AC for 2 h and then infected with RSV for 24 h. Following by fixation in 4% PFA, permeabilization in 0.01% Triton X-100 (BioFroxx, China) and blockade with 5% BSA, the cells were cultivated overnight with p-MLKL. FITC-conjugated secondary antibody and DAPI were utilized to label the first antibody and counterstain the nuclear respectively. Images were viewed *via* fluorescence microscope (Carl Zeiss, Germany).

### Gas chromatography-mass spectrometry measurement

50 µL serum specimen was blended in 200 µL methyl alcohol involving 1,2-^13^C-myristic acid and afterwards vortexed and centrifuged. Then 100 µL of supernatant was moved to another tube and subjected to evaporation. Samples were reconstituted in 30 µL of 10 mg/ml methoxyamine pyridine and oscillated for 1.5 h with the speed of 300 rpm at 30°C. 30 µL of BSTFA was supplemented and oscillated for 0.5 h at 37°C. After derivation and centrifugation, 50 µL of supernate was prepared for GC-MS analyses. Metabonomic analyses were conducted via Trace 1,310 gas chromatography and TSQ 8000 triple-quadrupole mass spectrometry detector (Thermo Fisher Scientific, USA) under the same conditions described before (Zhang H. R. et al., 2019). NIST Database (https://www.nist.gov/) and MS-DIAL (v.4.10) software were used to convert format, process and analyze data. The significantly differential metabolites were identified based on the threshold of fold change (FC) > 1.2 (or <0.83) and *p* < 0.05. The quantity of selected metabolites in each of these two groups was reflected *via* a venn graph. In addition, partial least squares discriminant analysis (PLS-DA) and enrichment analyses were conducted in MetaboAnalyst5.0 (http://www.metaboanalyst.ca/).

### Statistical analysis

GraphPad Prism 8.0 software (San Diego, USA) was utilized to conduct one-way analysis of variance (ANOVA) and Tukey’s post hoc test, in which data were described as mean ± standard deviation (SD), and *p* < 0.05 was considered statistically significant.

## Results

### AC mitigated lung injury and inhibited virus replication in RSV-infected mice

To investigate the effects of AC on RSV-induced lung injury, mice undergoing RSV infection were treated with AC, and ribavirin was used as a positive control drug (Figure 1B). Lung slices stained with H&E were used to evaluate pathological variations. As expected, lung tissues from RSV-infected mice presented severe inflammatory cell infiltration, oedema, reinforced interstitial congestion, and thicker alveolar walls, but these pathologic variations were significantly mitigated by AC and ribavirin (Figure 1C). To investigate whether AC could defend against RSV infection, the viral load in lung tissue was determined via plaque assay 4 days after RSV infection. As shown in Figure 1D, there was a significant decrease in viral load in the RSV-infected mice treated with AC and ribavirin, compared with the untreated mice. RSV-F protein promotes the fusion of virus and host cell, and inhibition of RSV-F can block virus replication. Consistently, the mRNA and protein levels of RSV-F were significantly decreased by AC (Figures 1E–G). Overall, these data suggested that AC effectively improved lung injury and inhibited virus replication effectively in RSV-infected mice.

### AC reduced cytokine expression in RSV-infected mice

**FIGURE 2:**
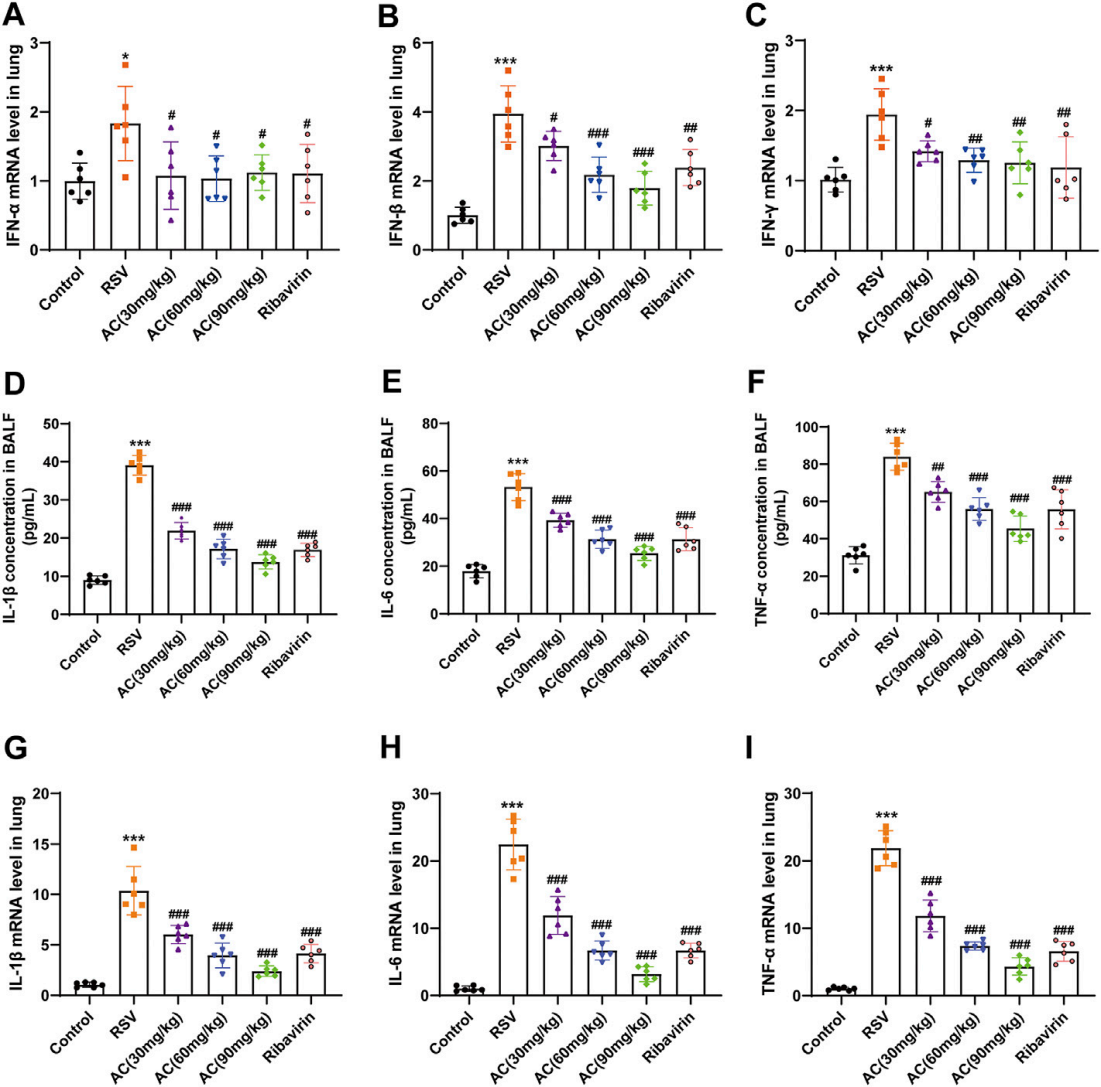
AC reduced cytokine expression in RSV-infected mice. **(A–C)** The mRNA levels of IFN-α, IFN-β and IFN-γ in lung tissues. **(D–F)** The contents of IL-1β, IL-6, and TNF-α in BALF. **(G–I)** The mRNA levels of IL-1β, IL-6, and TNF-α in lung tissues. Data are described as the mean ± SD (n = 6). *p < 0.05, ***p < 0.001, compared to the controls. ^#^ *p* < 0.05, ^##^ *p* < 0.01, and ^###^ *p* < 0.001 vs. the RSV group.

In the early stage of viral infection, innate immune signal is the first-line defense for virus clearance, and it also regulates the secretion of cytokines that mainly lead to inflammatory injury. Thus, we first assessed the effects of AC on IFN-α, IFN-β and IFN-γ mRNA expression in RSV-infected mice. RSV led to significant induction of type I and II IFN production in the lungs, all of which were suppressed by AC and ribavirin (Figures 2A–C). Also, IL-1β, IL-6, and TNF-α contents in BALF were reduced significantly in an AC concentration-dependent manner (Figures 2D–F). Consistently, the mRNA levels of IL-1β, IL-6, and TNF-α were significantly decreased by AC and ribavirin (Figures 2G–I). Collectively, these results indicated that AC might be a potent modulator of innate immune and inflammatory response.

### AC inhibited HMGB1 release and NF-κB activation both *in vivo* and *in vitro*

**FIGURE 3:**
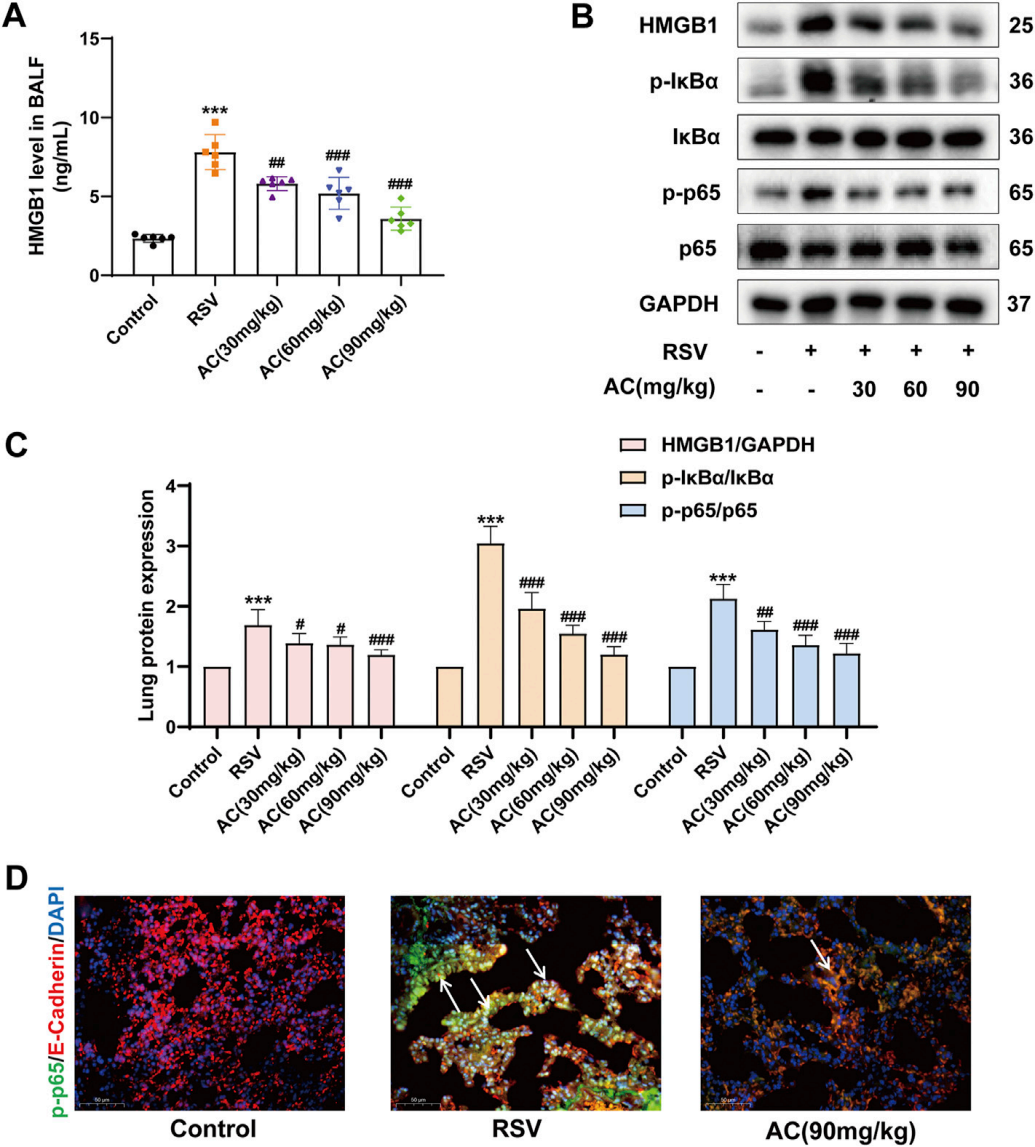
AC inhibited HMGB1 release and NF-κB stimulation in RSV-infected mice. **(A)** HMGB1 level in BALF (n = 6). **(B,C)** Western blot results and relative density analyses of HMGB1, p-IκBα/IκBα, and p-p65/p65 (n = 4). **(D)** Lung sections were stained with anti-E-cadherin (red) and anti-p-p65 (green) antibodies. The white arrow indicated the overlay panels. Scale bar, 50 μm. Data are described as the mean ± SD. ***p < 0.001, compared to the controls. ^#^ *p* < 0.05, ^##^ *p* < 0.01, and ^###^ *p* < 0.001 vs. the RSV group.

**FIGURE 4:**
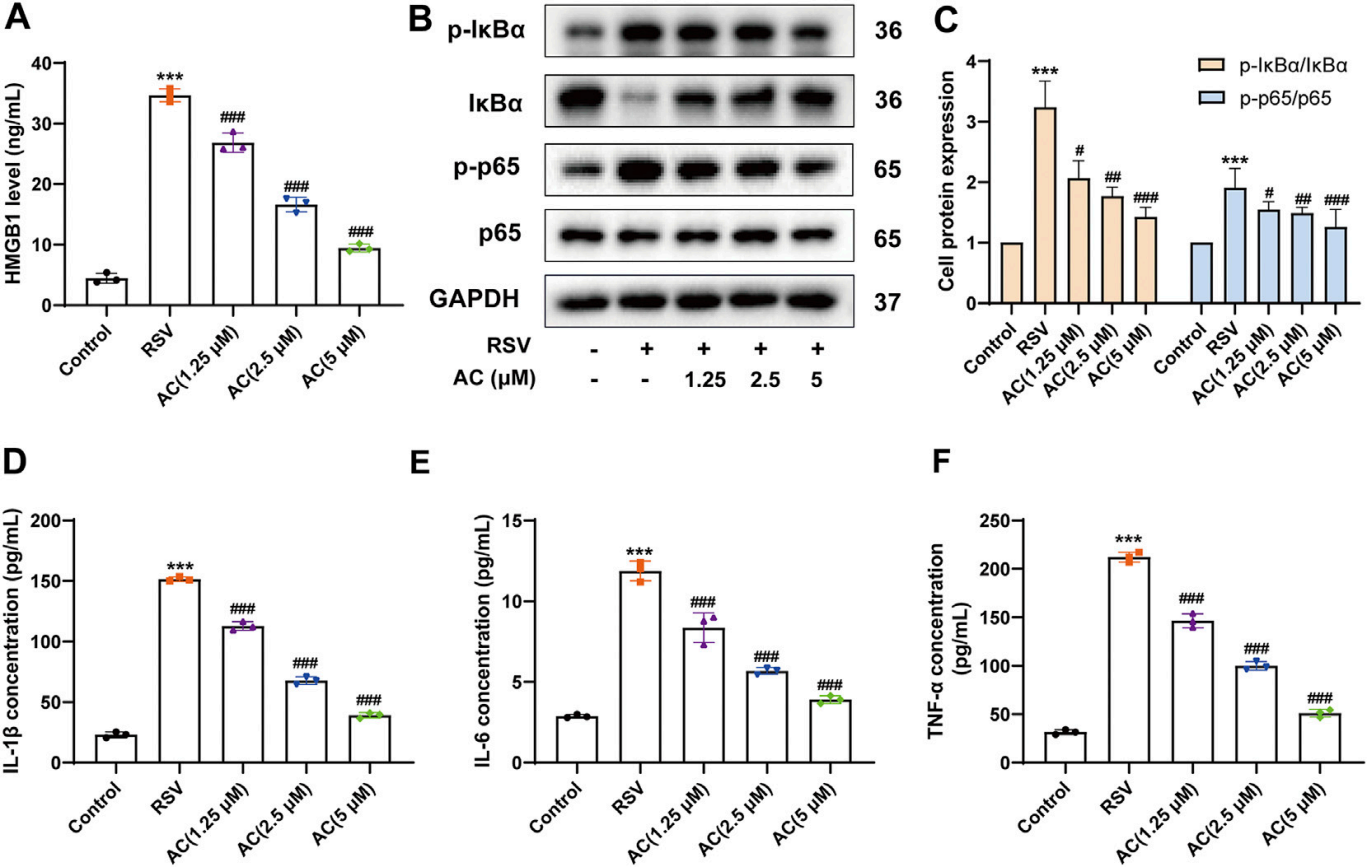
AC reduced HMGB1 release, NF-κB activation and inflammatory factors production in RSV-infected A549 cells. **(A)** HMGB1 level in the supernatant of A549 cells. **(B–C)** Western blot results and relative density analyses of p-IκBα/IκBα, and p-p65/p65 in the A549 cells. **(D–F)** The contents of IL-1β, IL-6, and TNF-α in the supernatant of A549 cells. Data are described as the mean ± SD (n = 3). ***p < 0.001, compared to the controls. ^#^ *p* < 0.05, ^##^ *p* < 0.01, and ^###^ *p* < 0.001 vs. the RSV group.

HMGB1 activates immune cells stimulated with RSV via the NF-κB pathway, which is pivotal for RSV-triggered pathogenesis (Rayavara et al., 2018; Niu et al., 2021). Therefore, it was investigated whether the anti-inflammatory potency of AC was attributable to the suppression of the HMGB1/NF-κB pathway. We found that RSV led to a significant increase of HMGB1 in BALF, which was reversed by AC treatment (Figure 3A). AC (1.25, 2.5, and 5 μM) also decreased the production of HMGB1 in RSV-infected A549 cells (Figure 4A).

Moreover, AC treatment reduced the HMGB1, p-IκBα and p-p65 levels in the lungs of RSV-infected mice (Figures 3B,C). We analyzed the level of p65 phosphorylation in lung epithelial cells of lung tissues from mice using immunofluorescence staining. RSV increased the intensity of p-p65-positive staining in E-cadherin-positive epithelial cells in lung tissues, which was reversed by AC treatment (Figure 3D). *In vitro*, AC decreased the expression of p-IκBα and p-p65 in RSV-infected A549 cells (Figures 4B,C). AC also suppressed the production of IL-1β, IL-6, and TNF-α in the supernatant of RSV-infected A549 cells (Figures 4D–F). These findings suggested that the protective role of AC was tightly related to the suppression of HMGB1 release and NF-κB stimulation.

### AC decreased RSV-induced necroptosis both *in vivo* and *in vitro*

**FIGURE 5:**
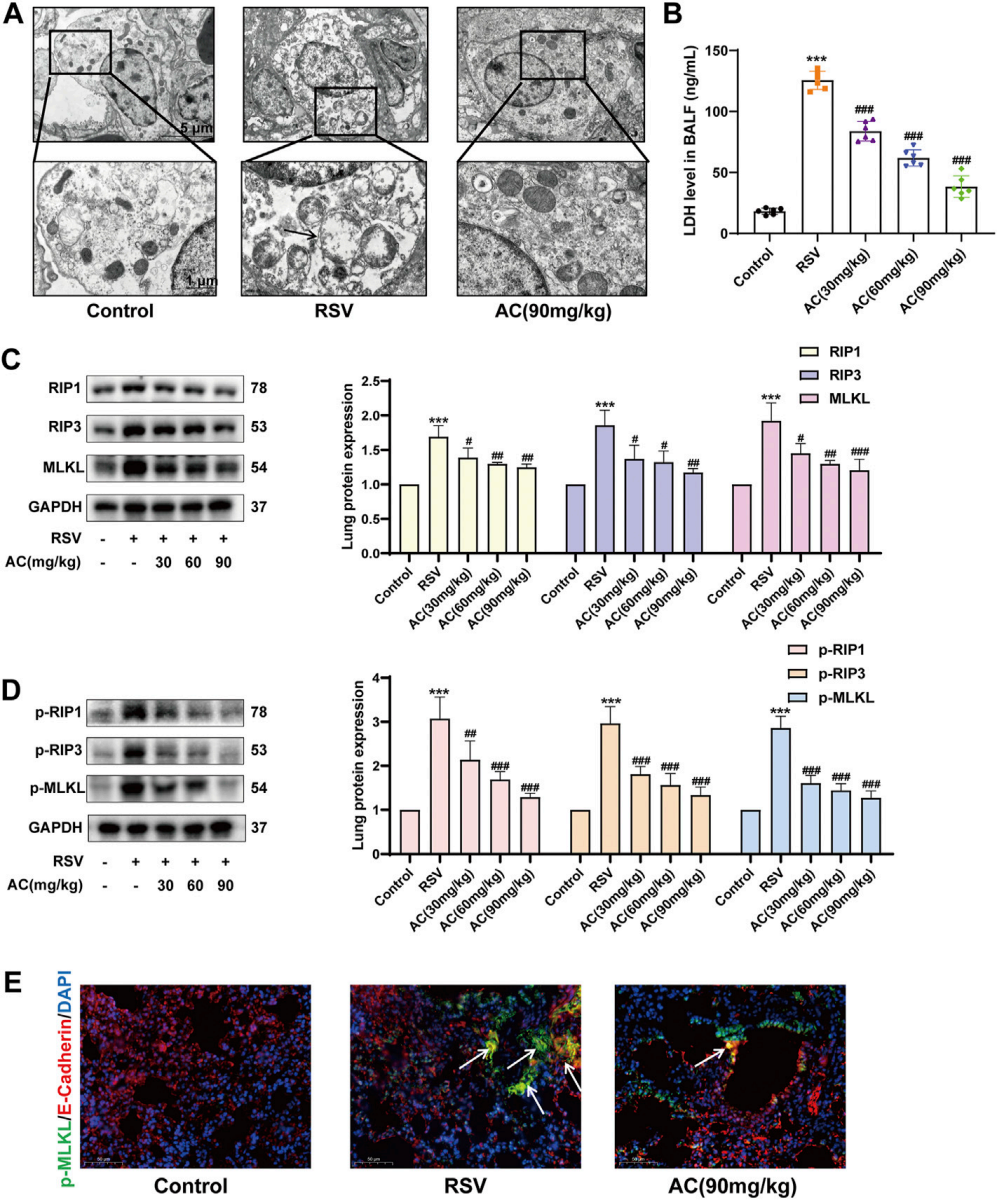
AC inhibited necroptosis *in vivo*. **(A)** TEM revealed AC could partially reverse mitochondrial damage in alveolar epithelial cells. The black arrow indicated plasma membrane rupture, mitochondrial swelling and vacuolation. **(B)** LDH level in BALF measured by ELISA. **(C,D)** Western blot results and relative density analyses of RIP1, RIP3, MLKL, p-RIP1, p-RIP3, and p-MLKL. **(E)** Lung sections were stained with anti-E-cadherin (red) and anti-p-MLKL (green) antibodies. The white arrow indicated the overlay panels. Scale bar, 50 μm. Data are described as the mean ± SD (n = 4). ***p < 0.001, compared to the controls. ^#^ *p* < 0.05, ^##^ *p* < 0.01, and ^###^ *p* < 0.001 vs. the RSV group.

Necroptosis is implicated in the outcomes of RSV infection and inhibition of necroptosis is crucial for attenuating disease progression (Simpson et al., 2020). TEM results showed that RSV induced mitochondrial swelling, a reduction of mitochondrial cristae, nuclear deformation, and plasma membrane rupture in alveolar epithelial cells. AC treatment partially reversed mitochondrial and cell membrane damage in RSV-infected mice (Figure 5A). LDH leakage can be used to verify the disruption of the cytoplasmic membrane, which is the main characteristic of necroptosis (Wang et al., 2021). As identified, the LDH content in BALF was remarkably reduced by AC administration (Figure 5B). Meanwhile, we evaluated the expression of RIP1, RIP3, and MLKL, which are pivotal for driving necroptotic cell death (Sauler et al., 2019). As shown in Figures 5C,D, RSV increased the expression of total and phosphorylated RIP1, RIP3, and MLKL. The daily AC treatment led to the downregulation of necroptosis-associated proteins in mice lung lysates. MLKL phosphorylation is an essential step to initiate necroptosis. The level of MLKL phosphorylation in lung epithelial cells of lung tissues from mice was detected via immunofluorescence staining. RSV increased the intensity of p-MLKL-positive staining in E-cadherin-positive epithelial cells in lung tissues, which was reversed by AC treatment (Figure 5E).

**FIGURE 6:**
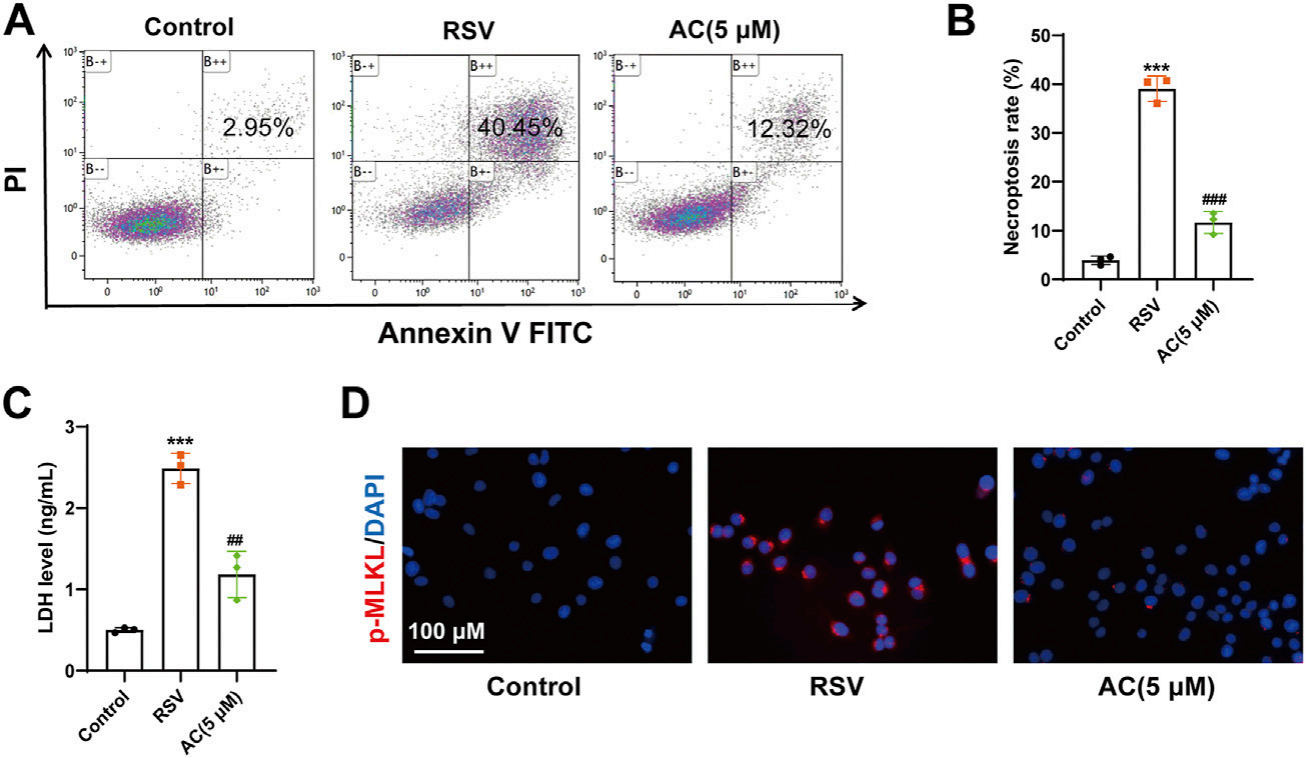
AC inhibited necroptosis *in vitro*. **(A,B)** Necroptotic proportions of A549 cells detected by flow cytometry. **(C)** LDH level in the supernatant of A549 cells. **(D)** Representative micrographs of pMLKL (red) in the A549 cells. Data are described as the mean ± SD (n = 3). ***p < 0.001, compared to the controls. ^##^ *p* < 0.01, and ^###^ *p* < 0.001 vs. the RSV group.

*In vitro*, a higher proportion of necroptotic cells was detected in the RSV-infected A549 cells compared to the controls, whereas AC significantly reduced the proportion of necroptotic cells (Figures 6A,B). AC also suppressed the level of LDH in RSV-infected A549 cells (Figure 6C). RSV significantly increased the intensity of p-MLKL staining in A549 cells, indicating the occurrence of necroptosis. After AC treatment, The increased expression of p-MLKL was reversed (Figure 6D). Taken together, these results indicated that AC reversed necroptosis in RSV-infected mice, as well as in RSV-infected A549 cells.

### AC relieved RSV-induced mitochondrial dysfunction both *in vivo* and *in vitro*

**FIGURE 7:**
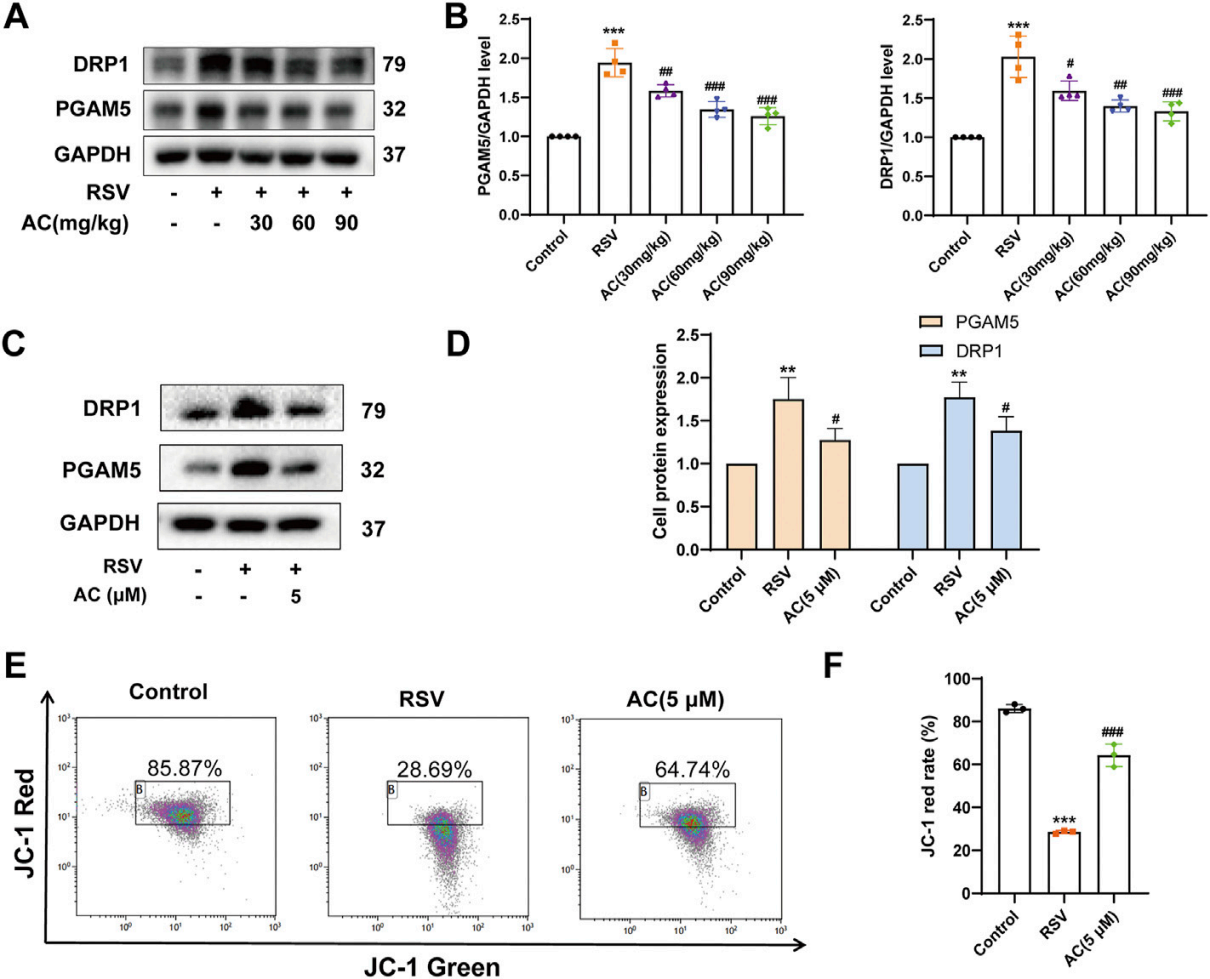
AC maintained mitochondrial function *in vivo* and *in vitro*. **(A,B)** Western blot results and relative density analyses of PGAM5 and DRP1 in lung tissue (n = 4). **(C,D)** Western blot results and relative density analyses of PGAM5 and DRP1 in A549 cells (n = 3). **(E,F)** MMP of A549 cells was detected by JC-1 staining (n = 3). Data are described as the mean ± SD. **p < 0.01, ***p < 0.001, compared to the controls. ^#^ *p* < 0.05, ^##^ *p* < 0.01, and ^###^ *p* < 0.001 vs. the RSV group.

We further identified the mitochondrial proteins PGAM5 and DRP1, which are vital for necroptosis execution (Wang et al., 2012). Compared to the controls, RSV increased the protein expression of PGAM5 and DRP1 in RSV-infected mice, which was inhibited by AC treatment (Figures 7A,B). *In vitro*, AC also decreased the levels of PGAM5 and DRP1 in RSV-infected A549 cells (Figures 7C,D).

As a cytosolic/outer mitochondrial membrane protein, DRP1 is pivotal for mitochondrial fission (Westermann, 2010). The stimulation of DRP1 can induce mitochondrial fragmentation, triggering mitochondrial function disorder. MMP level is a famous parameter for evaluating mitochondrial function. As presented in Figures 7E,F JC-1 red in RSV-infected A549 cells was reduced compared to the controls, suggesting an obvious reduction in MMP. While AC treatment led to an increase in higher JC-1 red, indicating that AC decreased mitochondrial depolarization by improving MMP. These results proved that AC could maintain the mitochondrial function.

### Metabonomic analyses of AC treatment in RSV-infected mice

**FIGURE 8:**
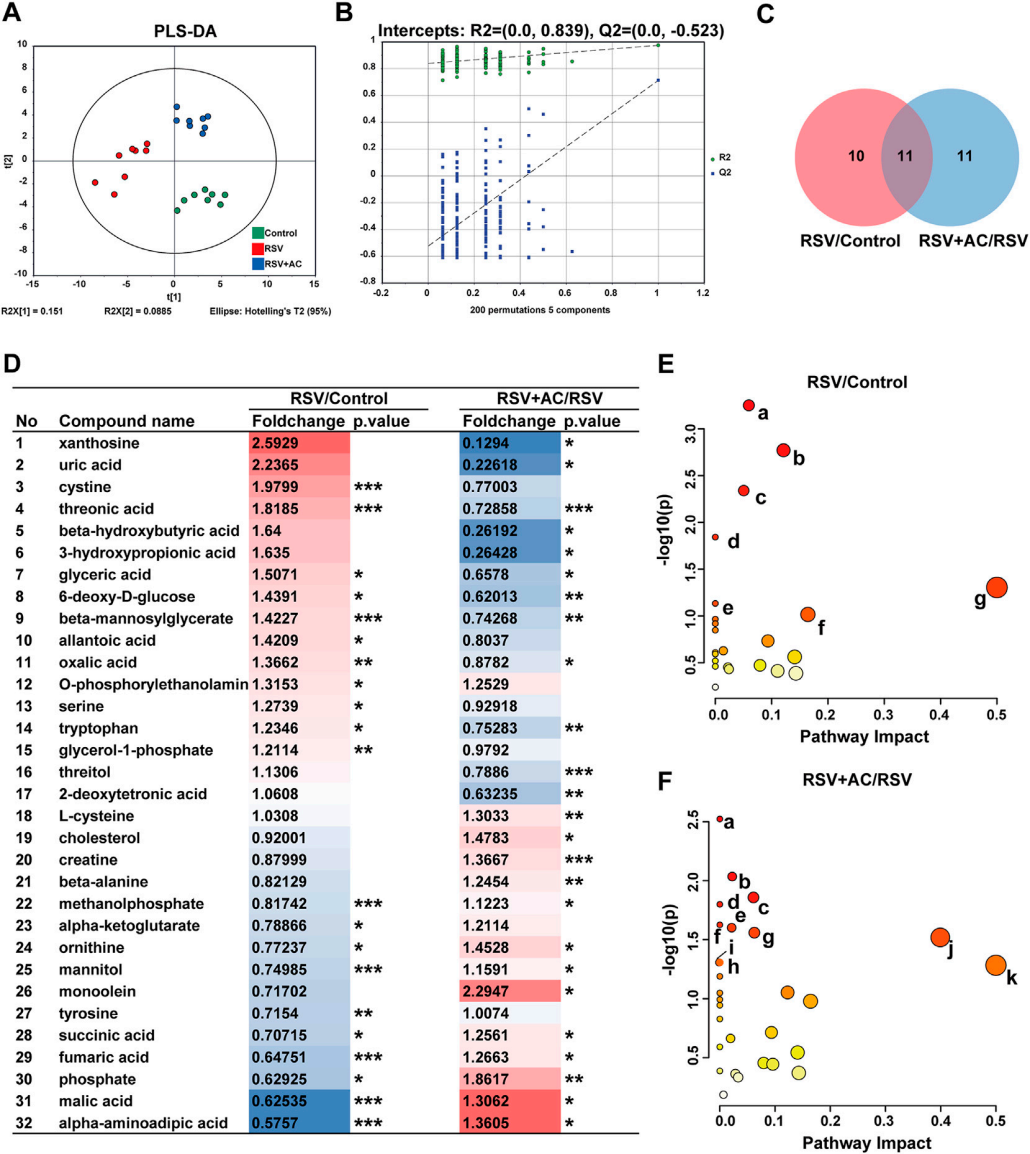
Metabonomic analyses of AC treatment in RSV-infected Mice. **(A)** PLS-DA plot of serum metabolites (*n* = 8). **(B)** PLS-DA permutation test graph (*n* =200). **(C)** Venn graph of differential metabolites in three groups. **(D)** The foldchange and *p*-values of 32 different metabolites. Fold change is color-coded, with red denoting up and blue indicating down-regulation. *p < 0.05, **p < 0.01, ***p < 0.001. **(E)** Pathway enrichment bubble diagram of RSV and control groups (a. Arginine biosynthesis, b. Citrate cycle, c. Alanine, aspartate and glutamate metabolism. d. Butanoate metabolism. e. D-glutamine and D-glutamate metabolism. f. Tyrosine metabolism. g. Phenylalanine, tyrosine and tryptophan biosynthesis). **(F)** Pathway enrichment bubble diagram of AC (90 mg/kg) and RSV groups (a. Propanoate metabolism. b. Glycine, serine and threonine metabolism. c. Arginine biosynthesis. d. Butanoate metabolism. e. Pantothenate and CoA biosynthesis. f. Aminoacyl-tRNA biosynthesis. g. Citrate cycle. h. Alanine, aspartate and glutamate metabolism. i. Alanine, aspartate and glutamate metabolism. j. beta-Alanine metabolism. k. Phenylalanine, tyrosine and tryptophan biosynthesis).

To demonstrate the system-wide mechanism of AC in treating RSV infection, we performed a metabolomic assay of serum using GC-MS. As shown in the PLS-DA patterns (Figure 8A), samples from the same group clustered together, while samples from different groups were well distinguished, reflecting that RSV infection and AC treatment caused obvious changes in metabolites. Following 200 permutation assays (Figure 8B), the lower values of the Q2 intercept revealed the stability of the models with a lower overfit risk.

Subsequently, based on the standard of FC > 1.2 or <0.83 and *p* < 0.05, 21 differential metabolites were identified in the RSV and control groups, 22 in the AC and RSV groups, and 11 in both comparisons (Figure 8C). As shown in Figure 8D, 6-deoxy-d-glucose, beta-mannosylglycerate, glyceric acid, threonic acid, and tryptophan were more highly expressed in the serum of RSV-infected mice, whereas alpha-aminoadipic acid, fumaric acid, malic acid, ornithine, phosphate, and succinic acid were less expressed. After AC therapy, all these metabolites were recovered at various degrees.

We further explored metabolic pathways using MetaboAnalyst to reveal the potential meaning of the varying metabolites. Substantial dramatically varied pathways in the RSV and AC groups were shown in Figures 8E,F. Among the shared enrichment pathways in the three groups, alanine, aspartate, and glutamate metabolism was one of the crucial metabolic components in the necroptosis pathway (KEGG pathway ID: map04217). In addition, necroptosis is also accompanied by mitochondrial damage and energy metabolic dysfunction (Chi et al., 2019). The main abnormal metabolic pathways related to energy metabolism were tricarboxylic acid (TCA) cycle, and pentose phosphate pathway. Our findings showed that the perturbations of major metabolites of AC therapy were related to variations in amino acid and energy metabolism.

## Discussion

Globally, RSV is the primary cause of childhood acute lower respiratory infections and hospital admissions (Shi et al., 2017). Great efforts have been made to characterize RSV pathogenesis and design novel treatment regimens (Elawar et al., 2021). Herein, it was shown that AC ameliorated the lung pathology markedly in RSV-infected mice. AC was first reported to exert protective effects against RSV-induced lung inflammation and necroptosis, and alveolar epithelium is one of the target cells. Further studies showed that AC also relieved RSV-induced mitochondrial dysfunction and regulated energy metabolism. Moreover, it was reported that no substantial toxicities were observed in mice treated with AC up to 800 mg/kg, indicating that AC is a relatively safe drug (Song et al., 2016).

AC has shown significant antiviral effects against various viral infections (Song et al., 2016). In this study, we found that despite reducing Type I and II IFN production, AC treatment led to a significant decrease in viral load and virus replication. IFN play important roles in the antiviral mechanism. However, aberrant IFN signaling during acute viral infections might induce an inflammatory response, leading to immune injury. Thus, appropriate regulation of the over-activated IFN signal did not affect the clearance of virus, and also reduced the highly expressed inflammatory cytokines. Our finding is in contrast to that AC prevented viral infection via inducing IFN-γ reported by Song et al. (Song et al., 2016). It is possible that the differences could be due to the virus species or the difference between *in vitro* and *in vivo* experiments.

The pro-inflammatory cytokines, such as TNF-α, IL-6, and IL-1β, are vital for the onset and progression of RSV, particularly in the early phases of inflammation, and persistent elevations of these cytokines usually indicate unsatisfactory prognosis (Tabarani et al., 2013; Vázquez et al., 2019). Previous studies reported that TNF-α, IL-1β, and IL-6 levels were elevated in RSV-induced lung inflammation (Lin et al., 2021). Herein, RSV notably increased the levels of TNF-α, IL-1β, and IL-6 in lung tissue and BALF. Interestingly, AC exhibited anti-inflammatory effects via suppressing their expression. HMGB1 is a vital inflammatory alarmin participating in the etiopathogenesis of a variety of viral infections and inflammatory diseases (Patel et al., 2018). Moreover, recombinant HMGB1 can deteriorate RSV-triggered lung inflammation, whereas anti-HMGB1 can remarkably decrease inflammatory reactions (Hosakote et al., 2016; Simpson et al., 2020). The outcomes herein also demonstrated that HMGB1 expression was remarkably regulated upward following RSV infection, but such an increase was suppressed by AC, revealing that AC could function as a HMGB1 suppressor. NF-κB is a vital transcription factor for HMGB1 biosynthesis; when stimulated, it is translocated to the nucleus and binds to several targeted genes, promoting inflammation and injury events (Chen et al., 2021). Our findings showed that AC treatment significantly inhibited NF-κB activation and HMGB1 release in the RSV-infected mice and A549 cells. Moreover, further investigations revealed that IκBα phosphorylation was also inhibited by AC, indicating that the anti-inflammatory effects of AC might be associated with the inhibition of the HMGB1/NF-κB pathway.

Cell death is closely associated with the bioactivities of living organisms. During RSV infection, necroptosis is deleterious to virus eradication, which further aggravates the immune pathology and possibly induces asthma; hence, inhibition of necroptosis may be a viable therapeutic strategy (Harker and Snelgrove, 2020). The elevated releases of HMGB1 and LDH were associated with the loss of plasma membrane integrity, shown by increased the Annexin V (+)/PI (+) double staining, all indicative of necroptosis. TEM analysis revealed that the ultrastructure of the RSV-infected lung was consistent with the characteristics of necroptosis. Simpson J et al. reported that RSV caused primary human airway epithelial cells necroptosis (Simpson et al., 2020). Our findings were similar to the results of this previous study, thus suggesting that RSV progression was associated with necroptosis. Although the molecular mechanisms involved in necroptosis remain elusive, the RIP1/RIP3/MLKL cascade may be responsible for mediating necroptosis. Administration of Nec-1 (a RIP1 inhibitor) could attenuate RSV-induced necroptosis and decrease HMGB1 release (Simpson et al., 2020). Our results showed that AC reversed RSV-induced necroptosis both *in vitro* and *in vivo*. It also decreased the inflammatory response, thereby alleviating lung injury, which might be one of the mechanisms of AC acting on RSV infection.

The necrosome complex (RIPK1/RIPK3/MLKL) is thought to cause necroptosis through inducing mitochondrial function disorder that involves various mechanisms, such as the activation of PGAM5 and DRP1 (Wang et al., 2012). PGAM5 is activated and moved to the outer mitochondria membrane, co-modulating DRP1, causing mitochondrial fission. PGAM5 and DRP1 play important roles in both necroptosis and mitochondrial function. Studies by She L et al. confirmed that the repression of PGAM5 or knockdown of PGAM5 evidently ameliorated ischemia/reperfusion injury, decreased cell necroptosis, and improved mitochondrial function (She et al., 2019). In the present study, MMP and ultrastructural analyse revealed that the mitochondria were impaired under RSV infection, whereas AC played a mitochondrial-protective role by attenuating these deteriorated effects. In addition, we observed that the increase of PGAM5 and DRP1 caused by RSV could be inhibited by AC administration. These outcomes suggested that AC might improve RSV-induced mitochondrial dysfunction by regulating PGAM5 and DRP1.

Moreover, necroptosis is closely related to the metabolic state of cells. Mitochondria are workhorses in terms of energy generation, and their impairment signifies the dysfunction of energy metabolism. According to previous studies, RSV modulated cellular metabolism towards the glycolytic and pentose phosphate pathways (Morris et al., 2020). Herein, our team delineated the serum metabolites of mice. As expected, alpha-ketoglutarate, fumaric acid, malic acid, and succinic acid levels in the TCA cycle were significantly reduced after RSV infection, indicating the disruption of the TCA cycle. As the most vital energy metabolism pathway, obstruction of the TCA cycle unavoidably induces the disruption of energy metabolism. Inadequate energy during RSV infection eventually reinforces other energy generation pathways, such as the creatine and ketone body metabolism, which could explain the increased beta-hydroxybutyrate levels in the RSV group. The above-mentioned findings demonstrated that the energy metabolism of RSV-infected mice was severely altered, and that AC might balance the energy metabolism. The TCA cycle is also the core of viral infection and replication along with viral etiopathogenesis and antiviral immunity (Sánchez-García et al., 2021). SARS-CoV-2 infection altered the metabolites of the TCA cycle, in which biomarkers of the oxidative TCA cycle such as fumarate, malate, citrate, and aconitate were decreased (Mullen et al., 2021). In addition, increasing evidence reveals that metabolism and inflammatory mechanisms are closely associated. Uric acid, derived from purine metabolism, was generated during cell injury with viral infections (Li et al., 2021). Uric acid could trigger ROS production and stimulated the release of NLRP3 inflammasome, which occurred following childhood wheezing or bronchitis and was related to IL-1β generation (Braga et al., 2017; Schuler et al., 2020). It was demonstrated that disrupting the uric acid pathway could ameliorate RSV immunopathology (Fonseca et al., 2020). Consistently, our metabolic analyses showed that the uric acid content was increased in the RSV group, but decreased in the AC group, indicating that AC might ameliorate lung inflammation by inhibiting uric acid production.

However, there are some limitations in our study. Firstly, it is necessary to study other cell lines, such as macrophages, to better evaluate the therapeutic effect of AC. Secondly, our study found that AC suppressed RSV-induced inflammation and necroptosis, as well as relieving mitochondrial dysfunction and regulating energy metabolism. But these events are induced in different time course with overlap and their internal relationship remains to be further explored.

## Conclusion

In conclusion, AC was first reported to exert protective effects against RSV-induced lung inflammation and necroptosis, as well as relieving mitochondrial dysfunction and regulating energy metabolism. Our study can offer a theoretical foundation for treating RSV and provide a more practical application value of AC.

## Data Availability

The original contributions presented in the study are included in the article/Supplementary Material, further inquiries can be directed to the corresponding authors.

## Glossary

AC: acteoside
RSV: respiratory syncytial virus
HMGB1: high-mobility group box 1
NF-κB: transcription factor nuclear factor-kappa B
RIP1: receptor interacting protein kinase 1
RIP3: receptor interacting protein kinase 3
MLKL: mixed-lineage kinase domain-like
PGAM5: phosphoglycerate mutase family member 5
DRP1: dynamin-related protein 1
ROS: reactive oxygen species
DAMP: damage-associated molecular patterns
HE: hematoxylin-eosin
IF: immunofluorescence
IHC: immunohistochemistry
MOI: multiplicity of infection
TEM: transmission electron microscopy
FITC: fluorescein isothiocyante
PI: propidiumiodide
MMP: mitochondrial membrane potential
BALF: bronchoalveolar lavage fluid
TNF-α: tumor necrosis alpha
IL-6: interleukin 6
IL-1β: interleukin 1β
LDH: lactate dehydrogenase
ELISA: enzyme-linked immunosorbent assay
RT-qPCR: real-time polymerase chain reaction
RSV-F: RSV fusion
GC-MS: gas chromatography-mass spectrometry
FC: fold change
PLS-DA: partial least squares discriminant analysis
SD: standard deviation
ANOVA: one-way analysis of variance
TCA: tricarboxylic acid
