# Supplementary material for: Acteoside attenuates RSV-induced lung injury by suppressing necroptosis and regulating metabolism
Source: Front Pharmacol. 2022 Aug 19;13:870928. doi: 10.3389/fphar.2022.870928 (PMC9437591; doi:10.3389/fphar.2022.870928)
Supplement: Supplementary file 1 [file DataSheet7.PDF]

Supplementary Material

Table S1 Sequences of the gene-specific primers.

Gene	FORWARD PRIMER (5'-3')	REVERSE PRIMER (5'-3')
β-actin	GTATCCTGACCCTGAAGTACC	TGAAGGTCTCAAACATGATCT
IL-1β	GCAACTGTTCCTGAACTCAACT	ATCTTTTGGGGTCCGTCAACT
IL-6	CTCCCAACAGACCTGTCTATAC	CCATTGCACAACTCTTTTCTCA
TNF-α	ATGTCTCAGCCTCTTCTCATTC	GCTTGTCACTCGAATTTTGAGA
RSV-F	TGAAAGTCCACCTCCTTACAGA	CCGGATAAAAAGAGTACGCTGG
IFN-α	AAGCCATCCCTGTCCTGAGTGAG	GCTGCTGGTGGAGGTCATTGC
IFN-β	CAGCTCCAAGAAAGGACGAAC	GGCAGTGTAACTCTTCTGCAT
IFN-γ	ATGAACGCTACACACTGCATC	CCATCCTTTTGCCAGTTCCTC
