# Supplementary figures and images for: Acteoside attenuates RSV-induced lung injury by suppressing necroptosis and regulating metabolism
Source: Front Pharmacol. 2022 Aug 19;13:870928. doi: 10.3389/fphar.2022.870928 (PMC9437591; doi:10.3389/fphar.2022.870928)

## RSV-F Immunohistochemistry

Control

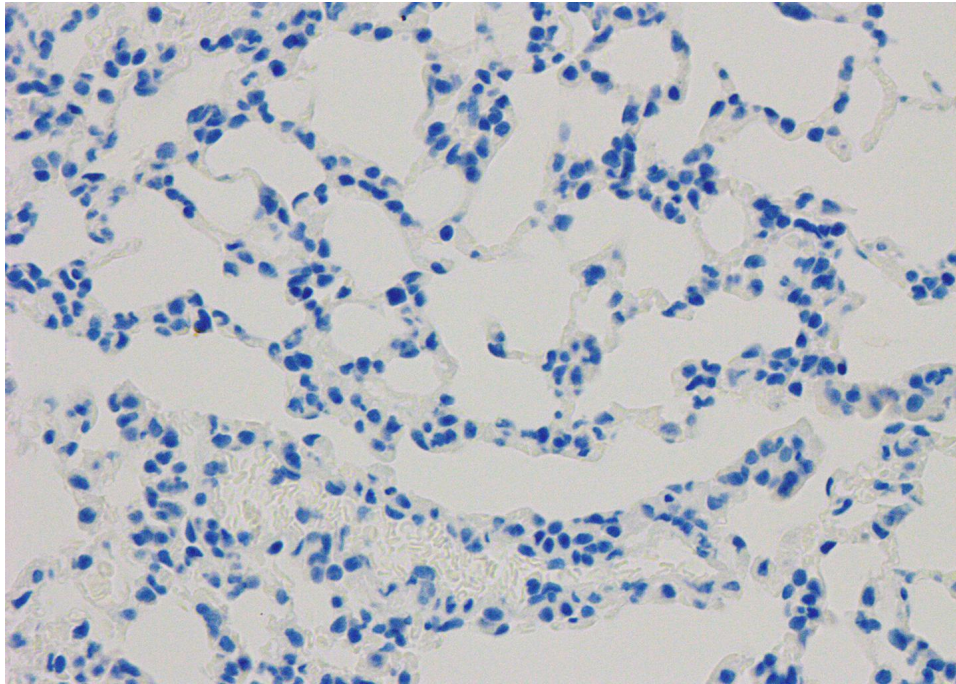

RSV

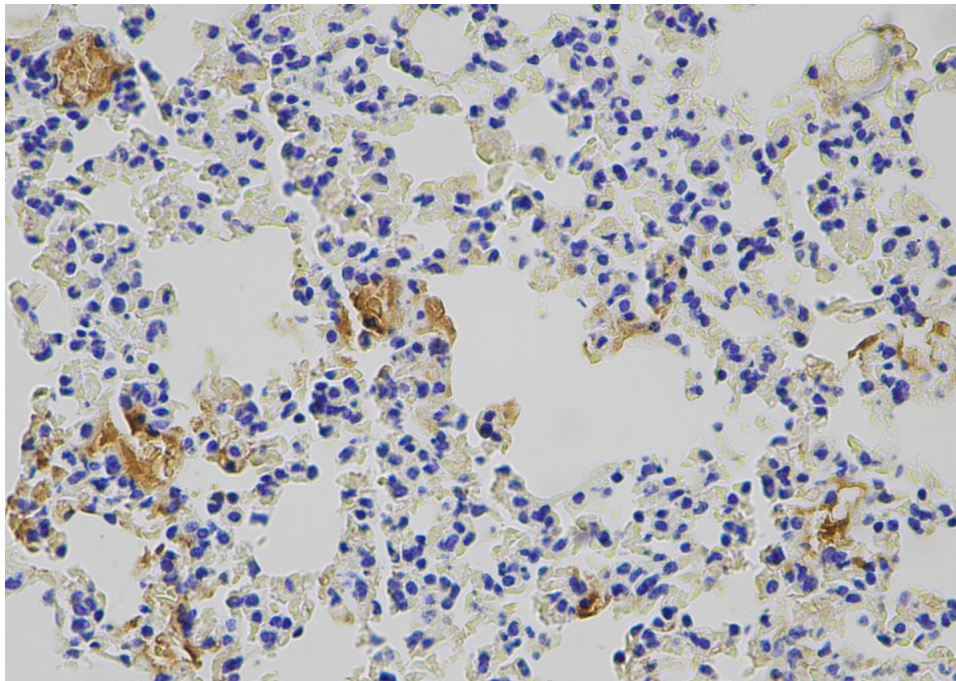

AC (30mg/kg)

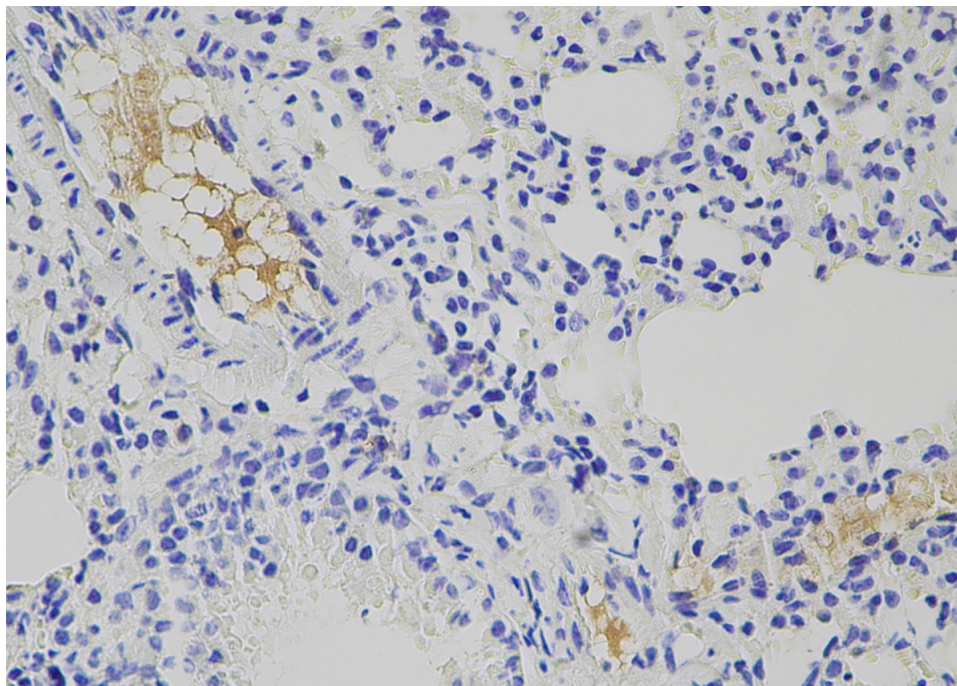

AC (60mg/kg)

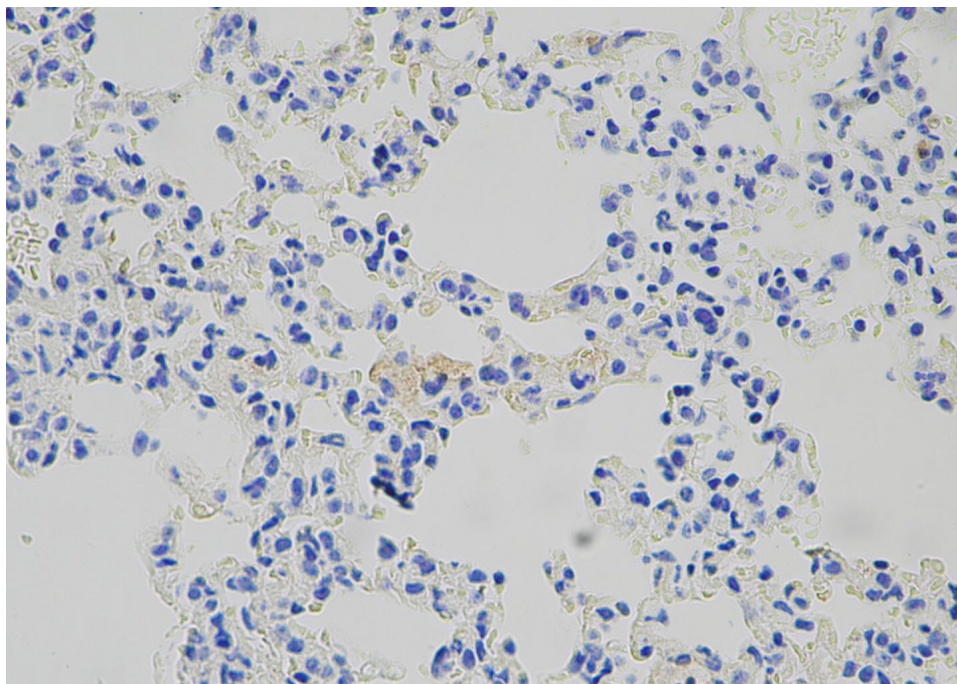

AC (90mg/kg)

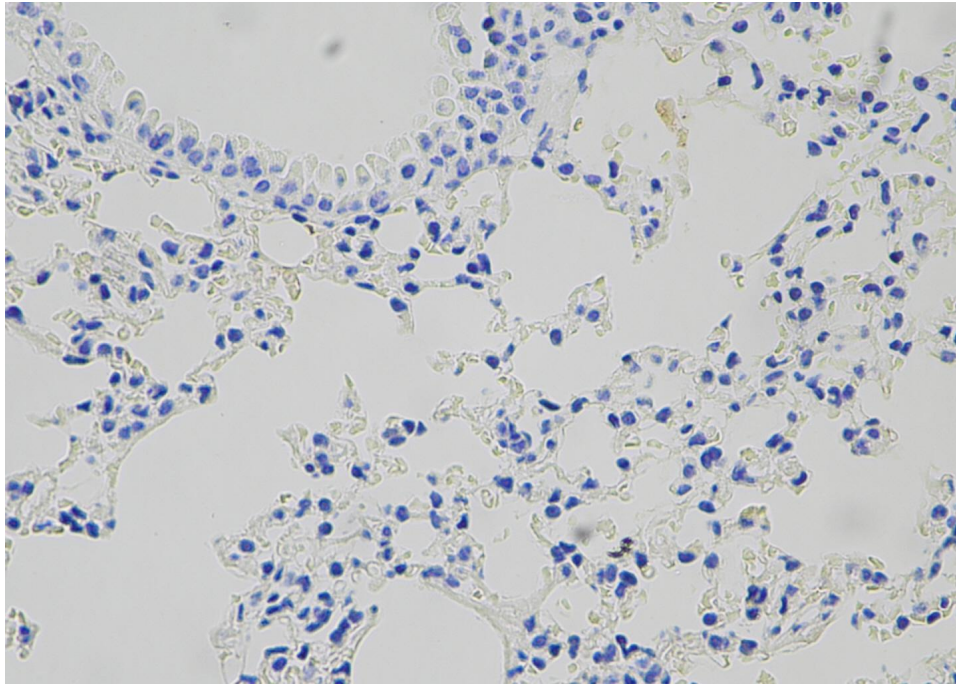

Ribavirin

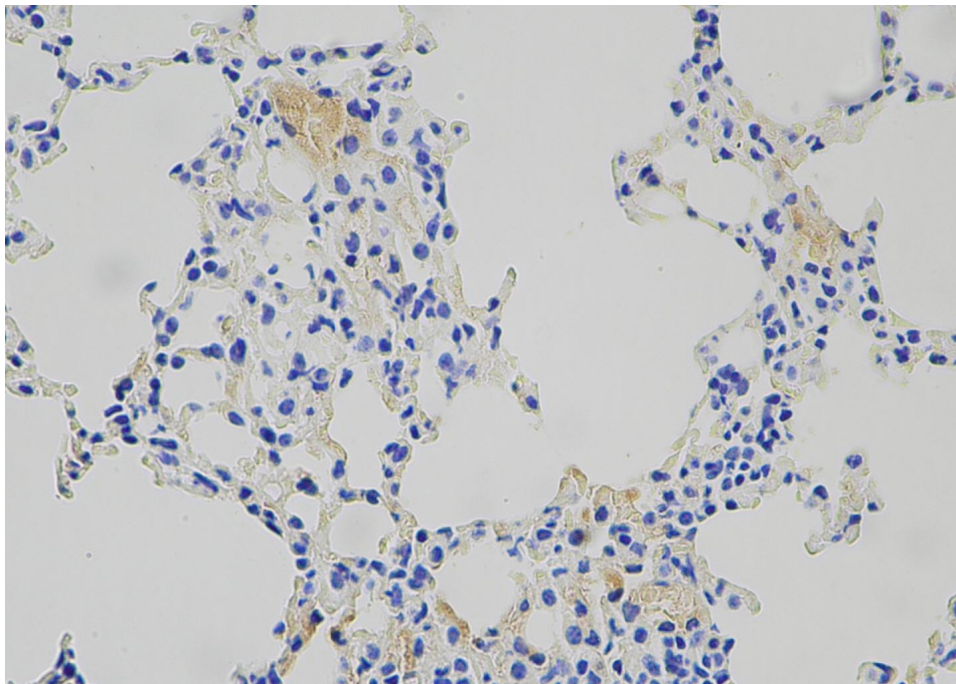

Supplement: Supplementary file 2 [file DataSheet2.PDF]

## Control

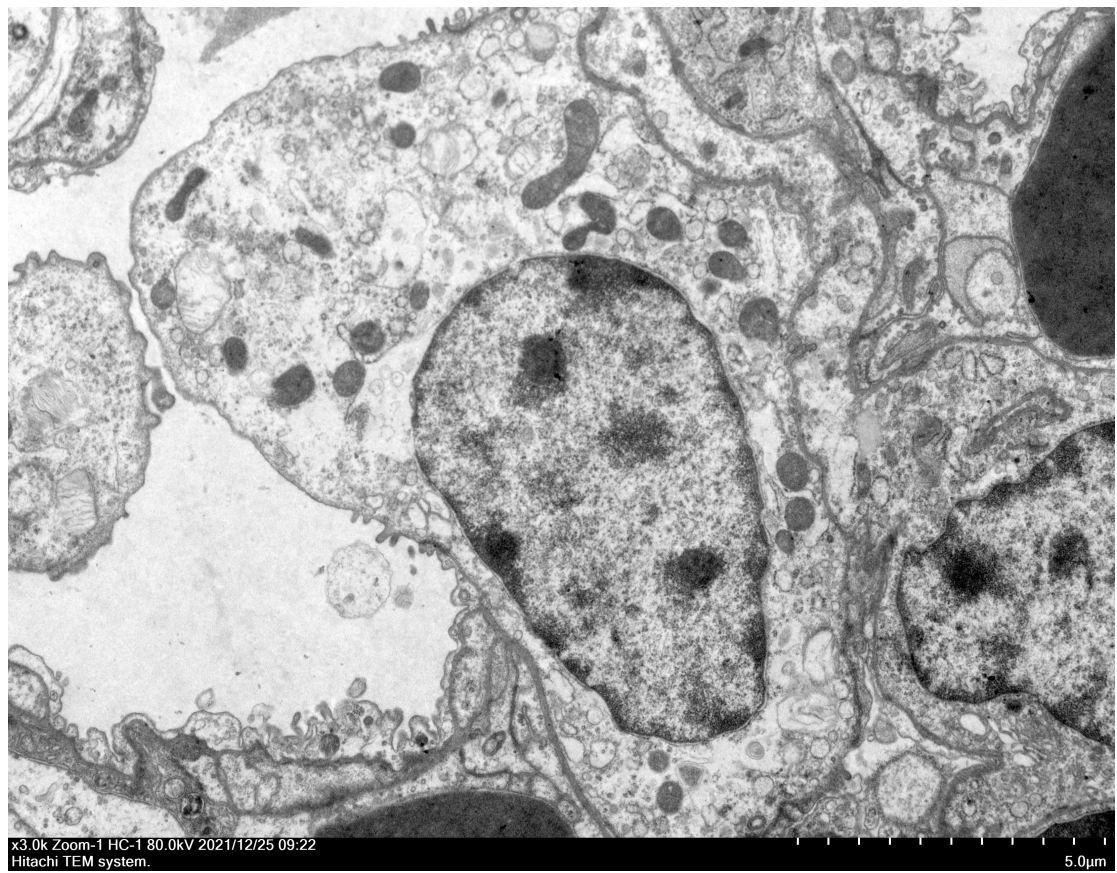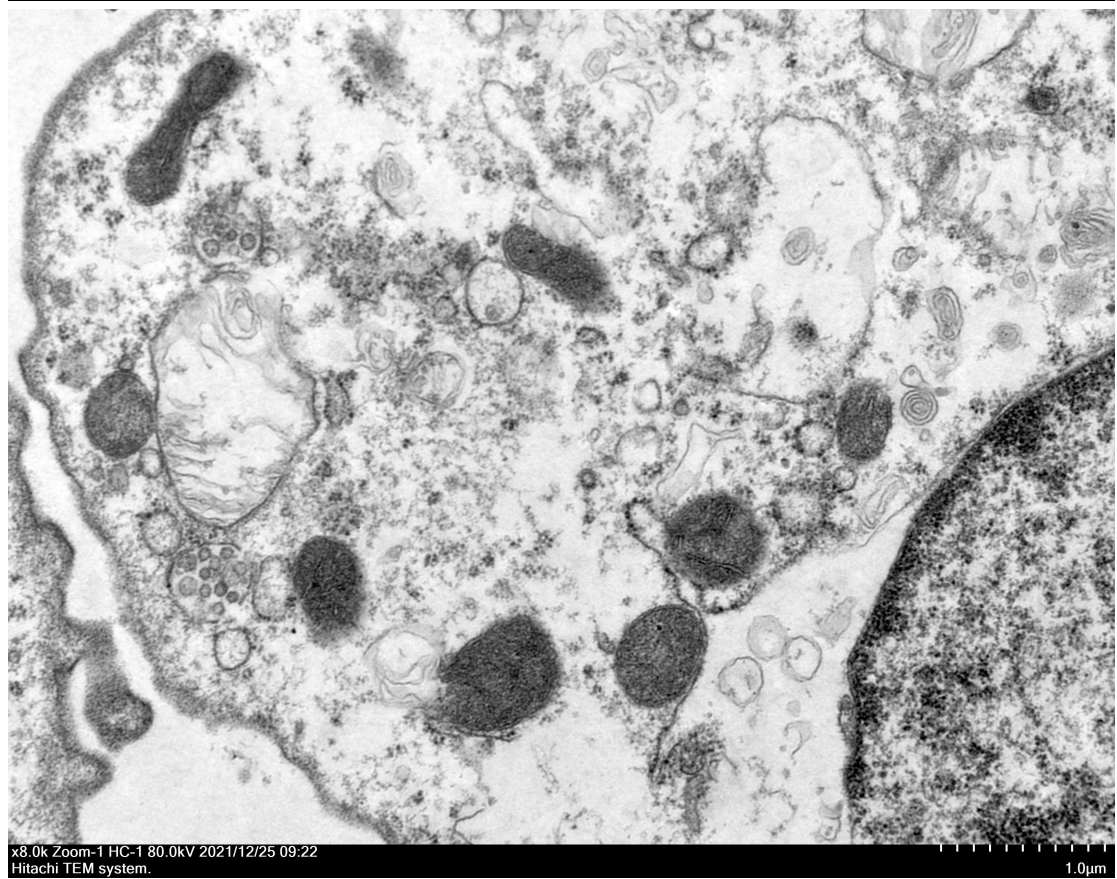

RSV

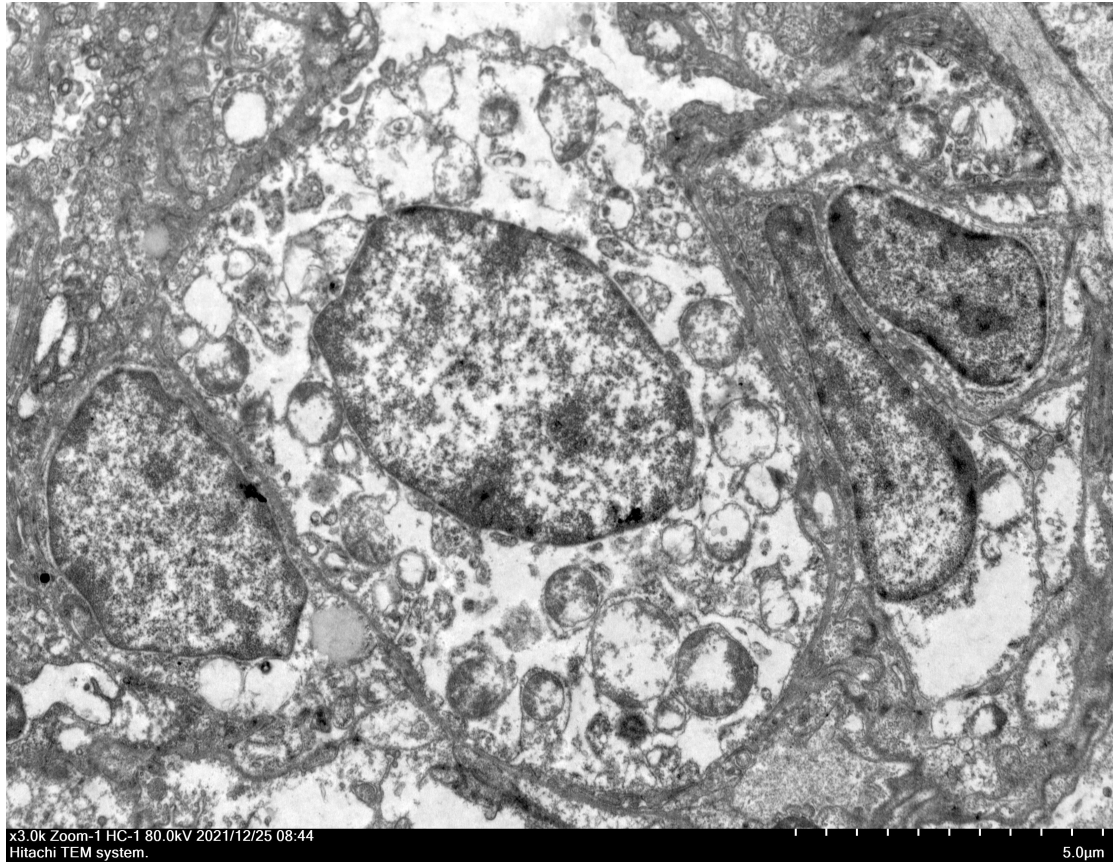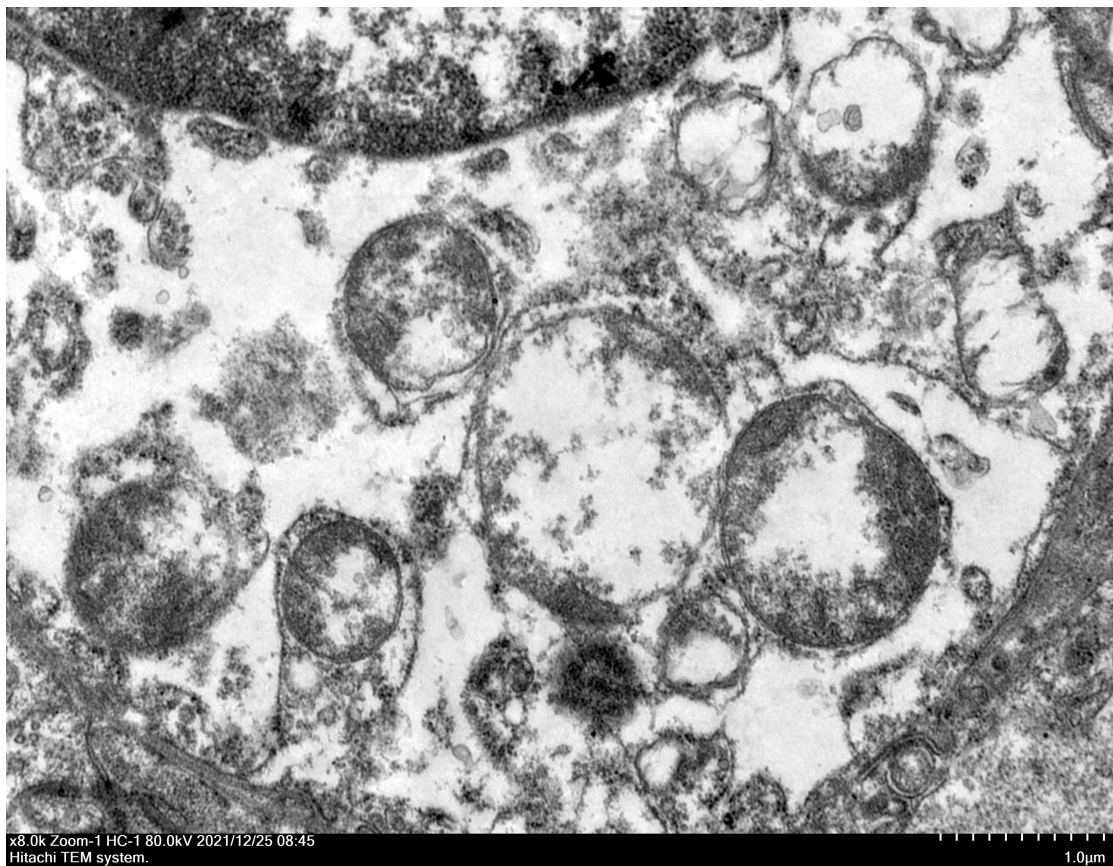

AC (90mg/kg)

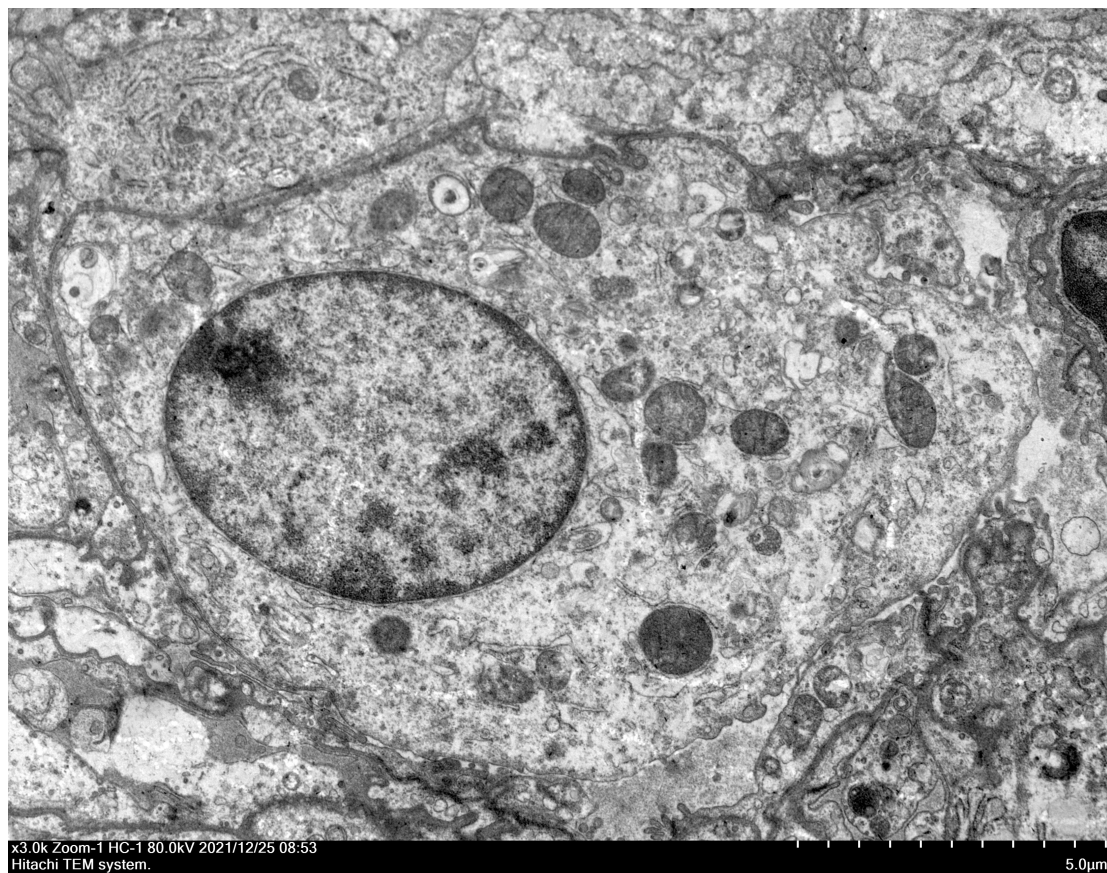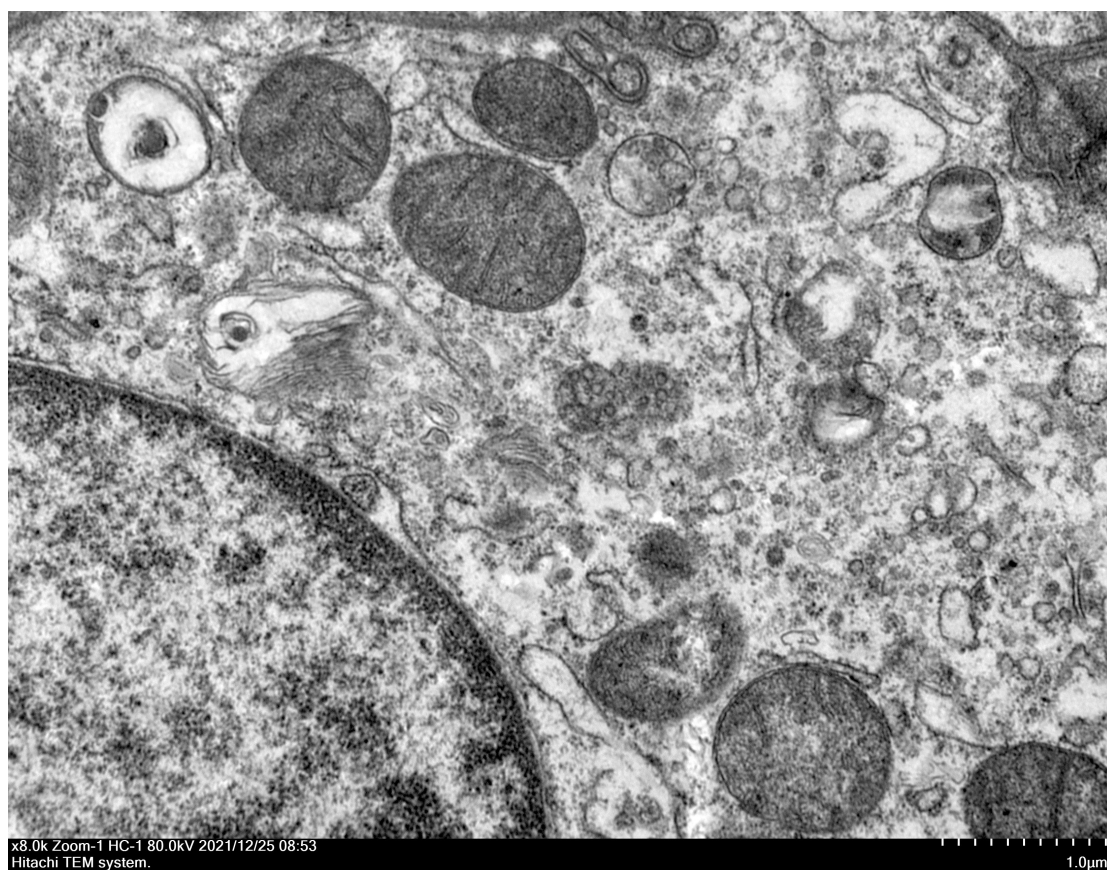

Supplement: Supplementary file 6 [file DataSheet3.PDF]

HE

Control

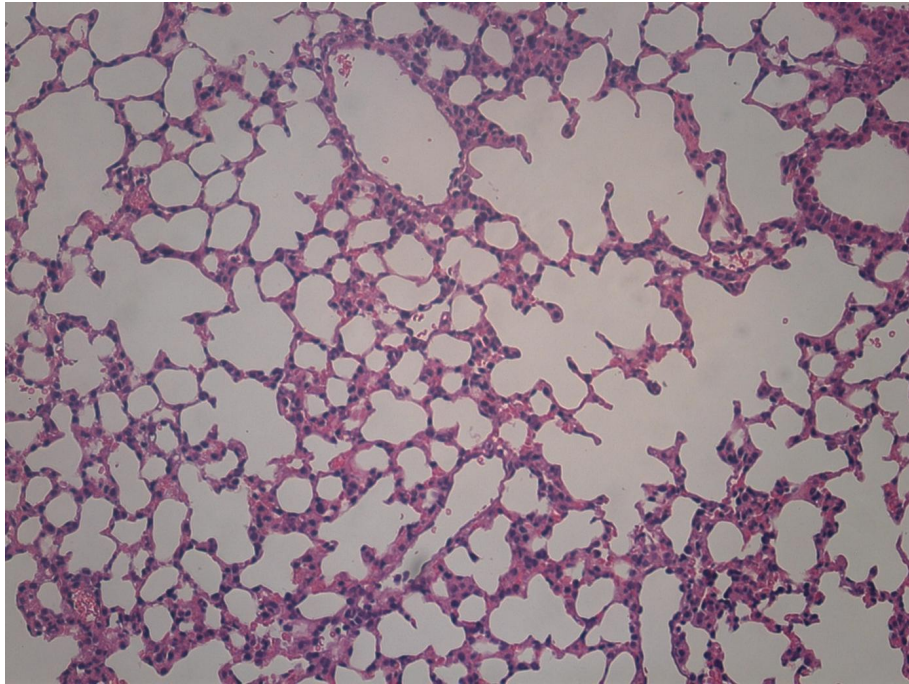

RSV

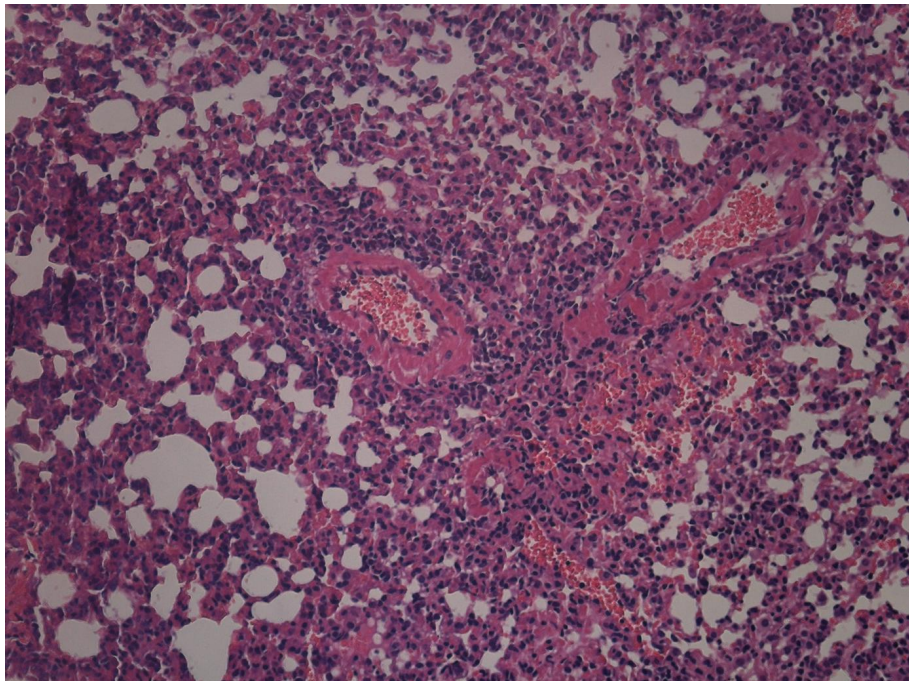

AC (30mg/kg)

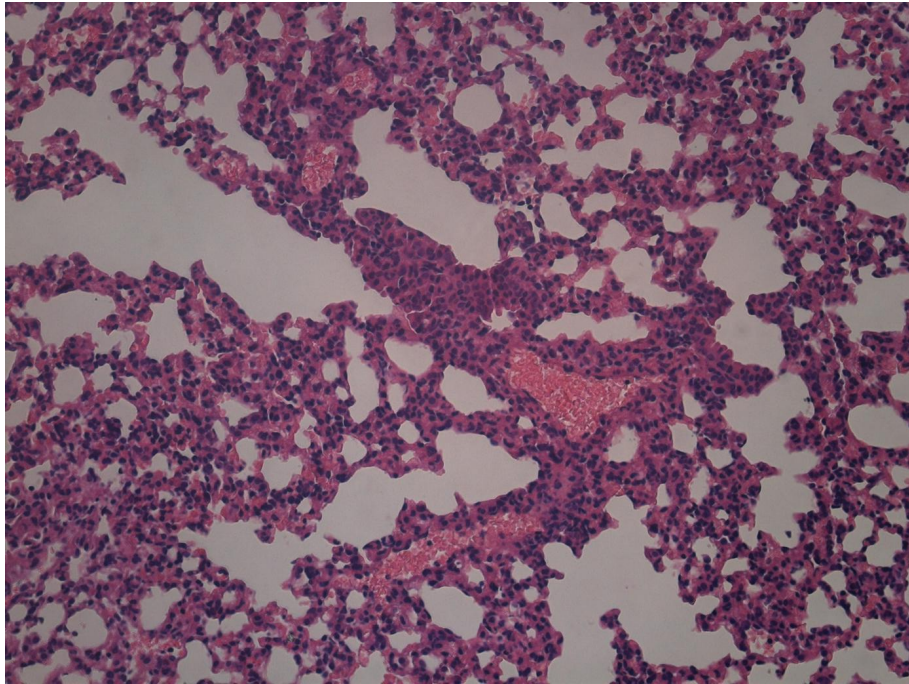

AC (60mg/kg)

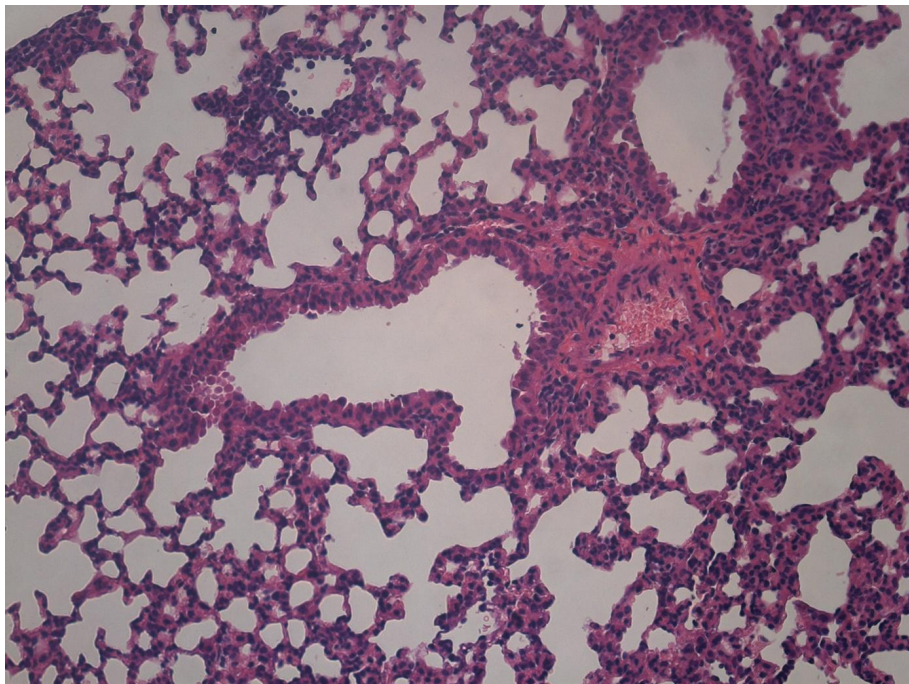

AC (90mg/kg)

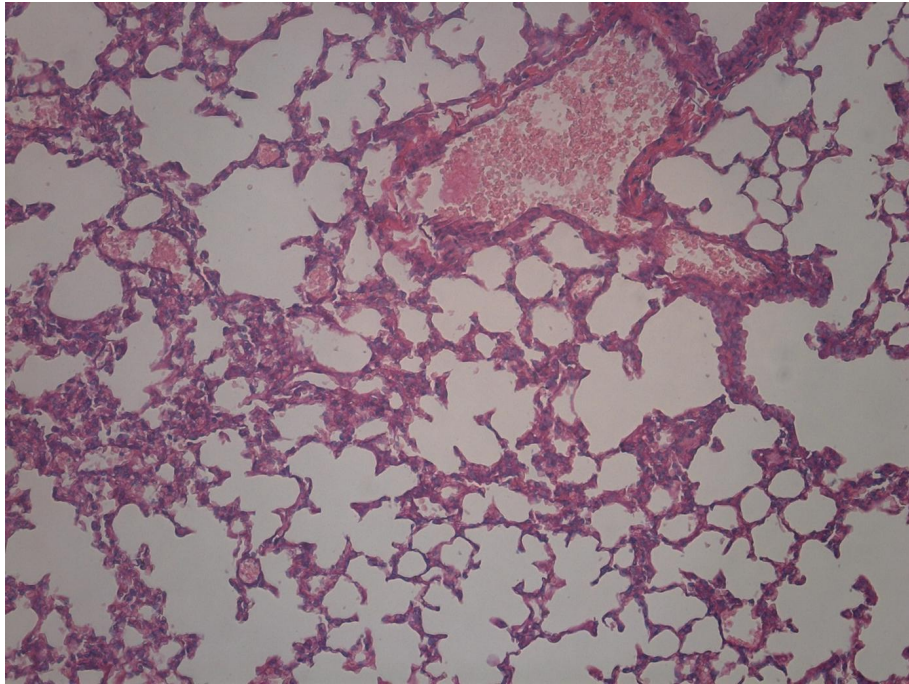

Ribavirin

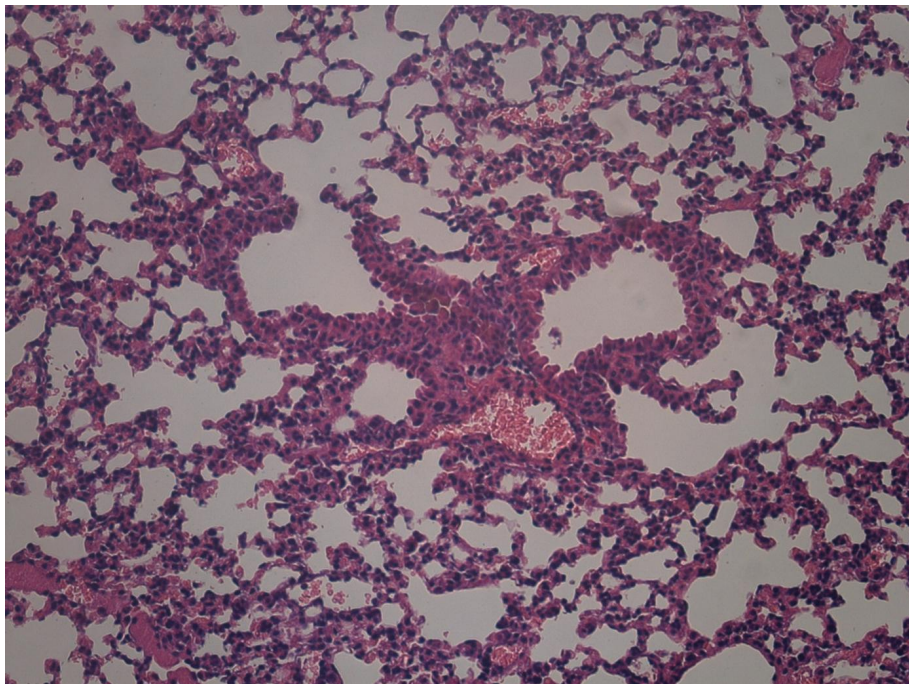

Supplement: Supplementary file 7 [file DataSheet1.PDF]
