# Supplementary material for: Acteoside attenuates RSV-induced lung injury by suppressing necroptosis and regulating metabolism
Source: Front Pharmacol. 2022 Aug 19;13:870928. doi: 10.3389/fphar.2022.870928 (PMC9437591; doi:10.3389/fphar.2022.870928)
Supplement: Supplementary file 3 [file DataSheet4.PDF]

Gating strategies for necroptosis flow assays

Control：

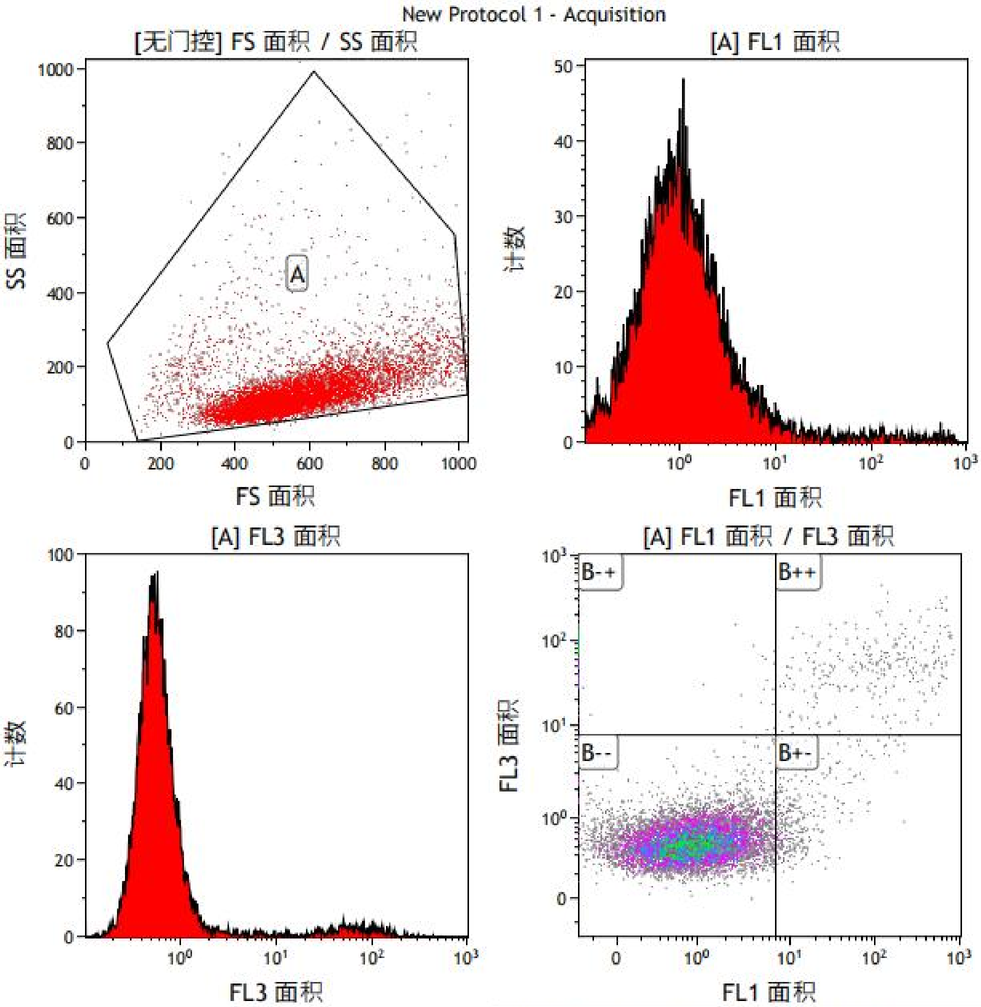

门控	数量	总计百分比	门控百分比
全部	10,151	91.57	100.00
B--	9,262	83.55	91.24
B-+	246	2.22	2.42
B+-	344	3.10	3.39
B++	299	2.70	2.95

RSV：

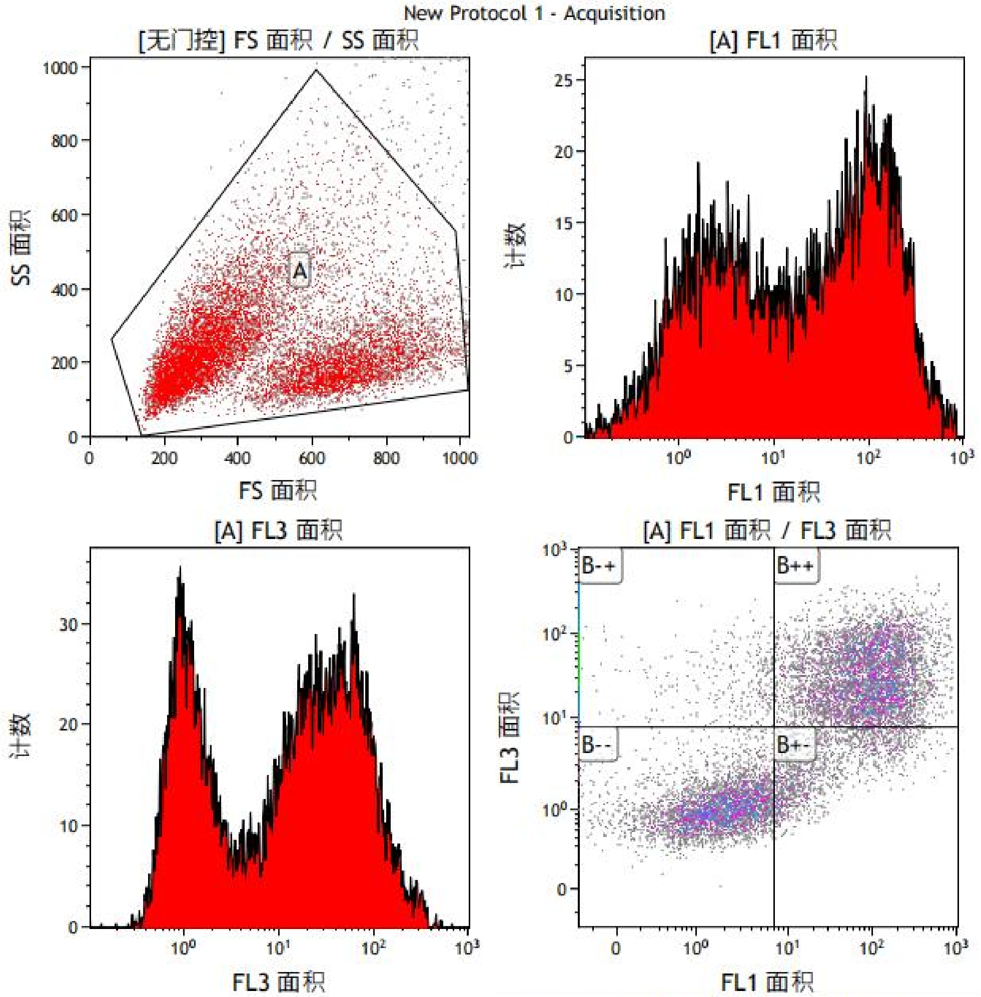

门控	数量	总计百分比	门控百分比
全部	12,001	93.06	100.00
B--	3,528	27.36	29.40
B-+	2,320	17.99	19.33
B+-	1,299	10.07	10.82
B++	4,854	37.64	40.45

AC (5 μM)

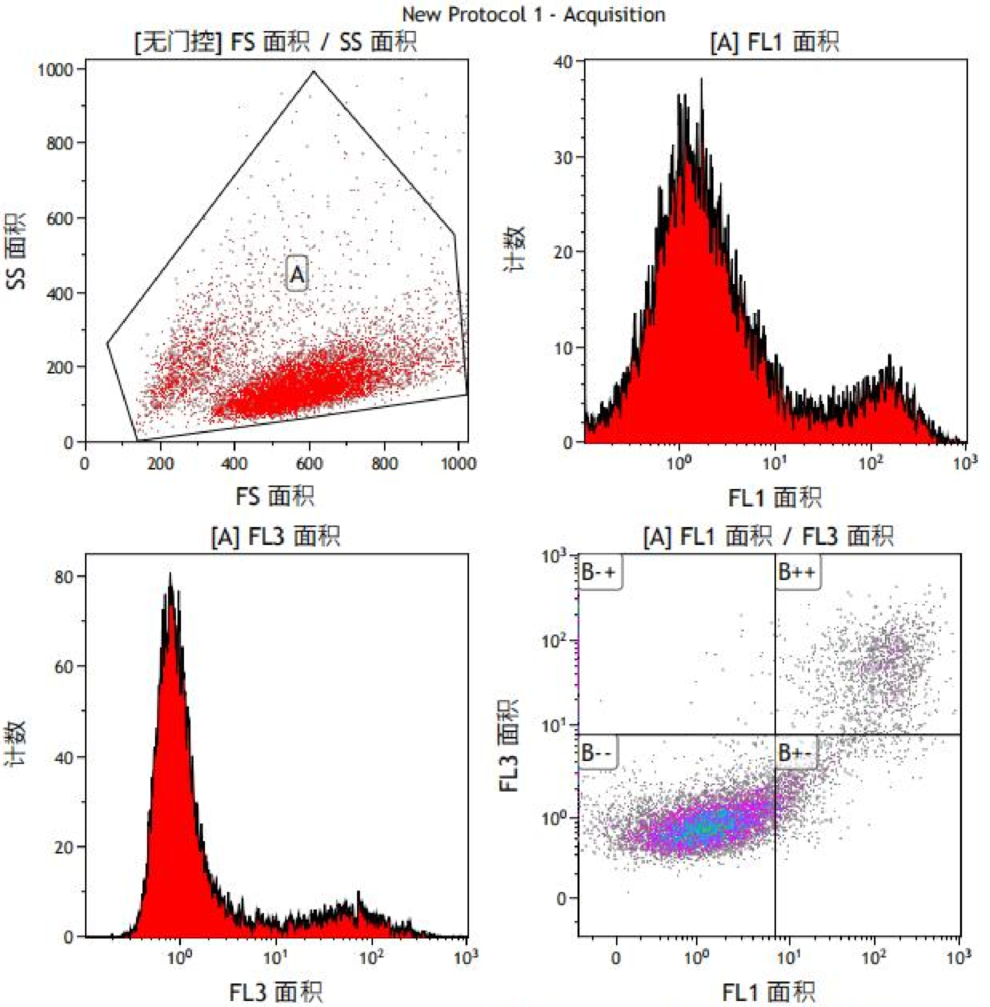

门控	数量	总计百分比	门控百分比
全部	10,372	96.47	100.00
B--	7,937	73.82	76.52
B-+	277	2.58	2.67
B+-	880	8.18	8.48
B++	1,278	11.89	12.32

Gating strategies for mitochondrial membrane potential flow assays

Control

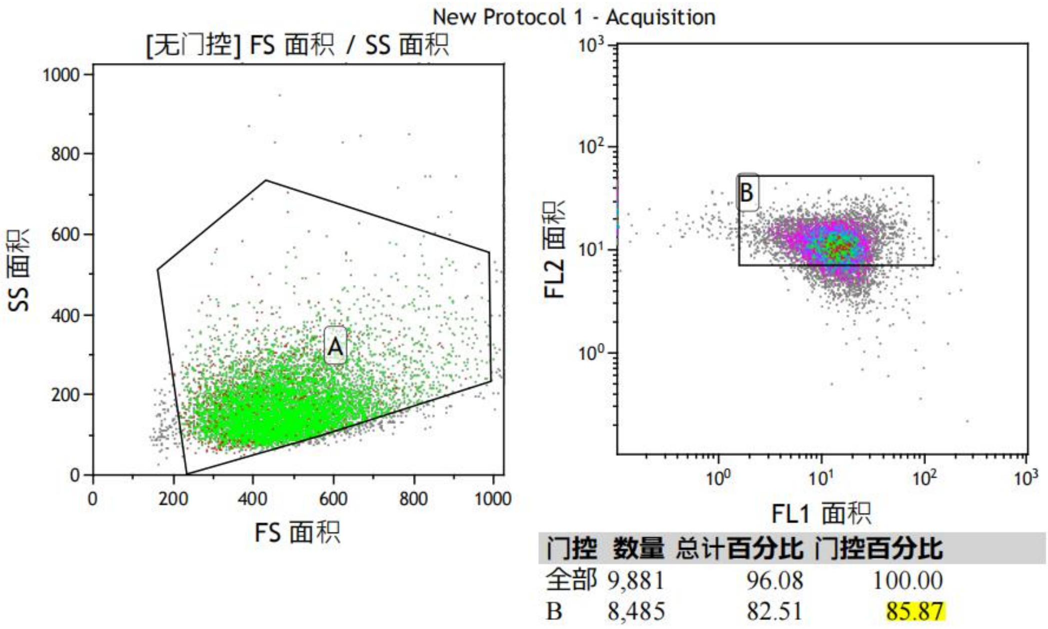

门控	数量	总计百分比	门控百分比
全部	9,881	96.08	100.00
B	8,485	82.51	85.87

RSV

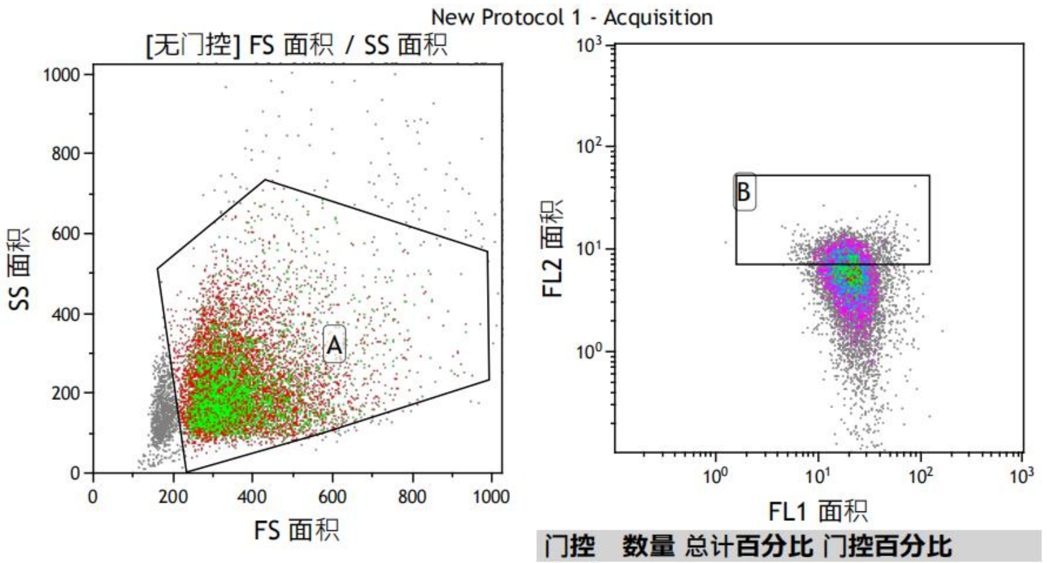

门控	数量	总计百分比	门控百分比
全部	10,130	89.02	100.00
B	2,906	25.54	28.69

AC (5 μM)

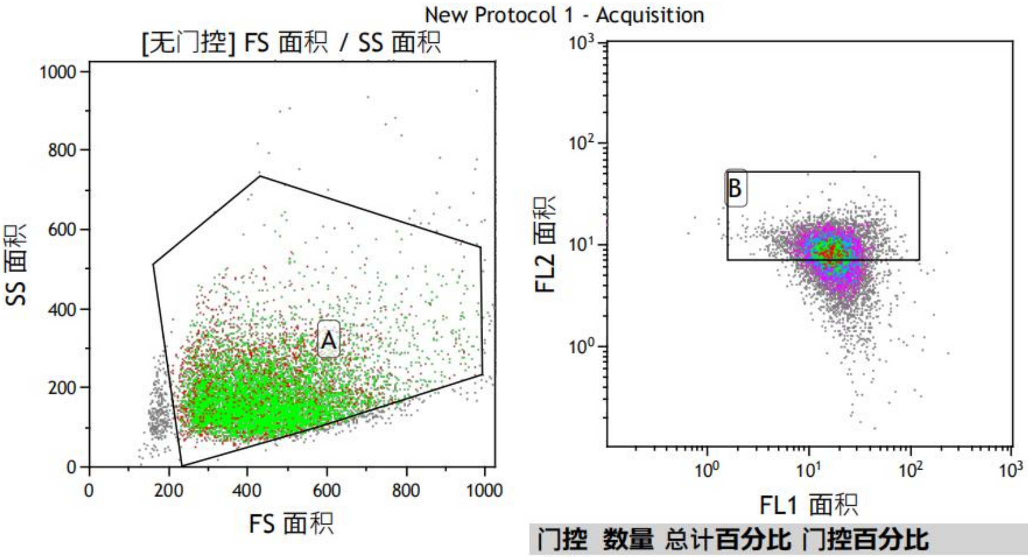

门控	数量	总计百分比	门控百分比
全部	9,892	94.16	100.00
B	6,404	60.96	64.74
