# Supplementary material for: Acteoside attenuates RSV-induced lung injury by suppressing necroptosis and regulating metabolism
Source: Front Pharmacol. 2022 Aug 19;13:870928. doi: 10.3389/fphar.2022.870928 (PMC9437591; doi:10.3389/fphar.2022.870928)

GAPDH in Figure3

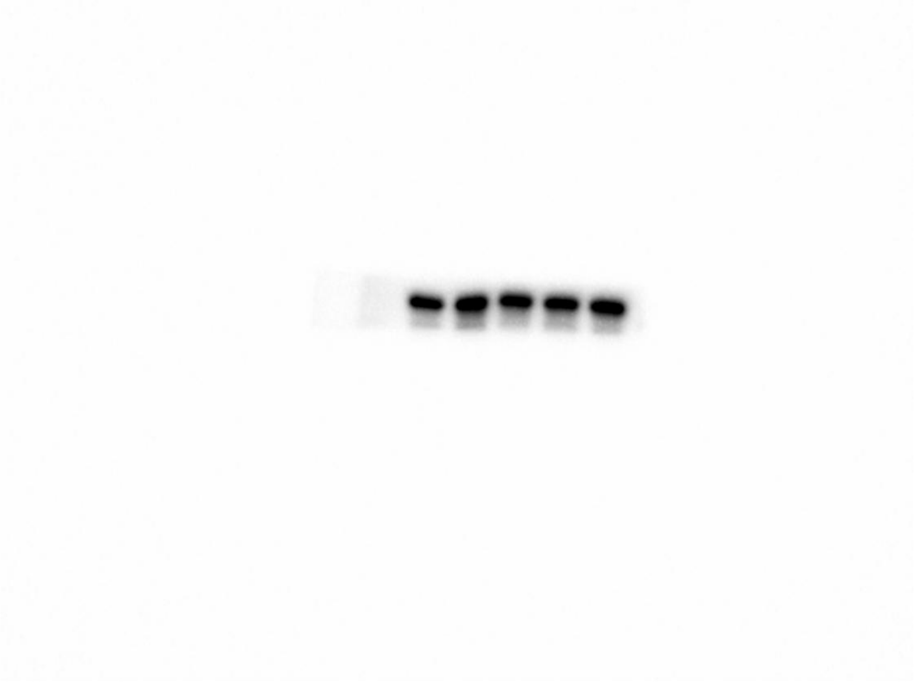

HMGB1 in Figure3

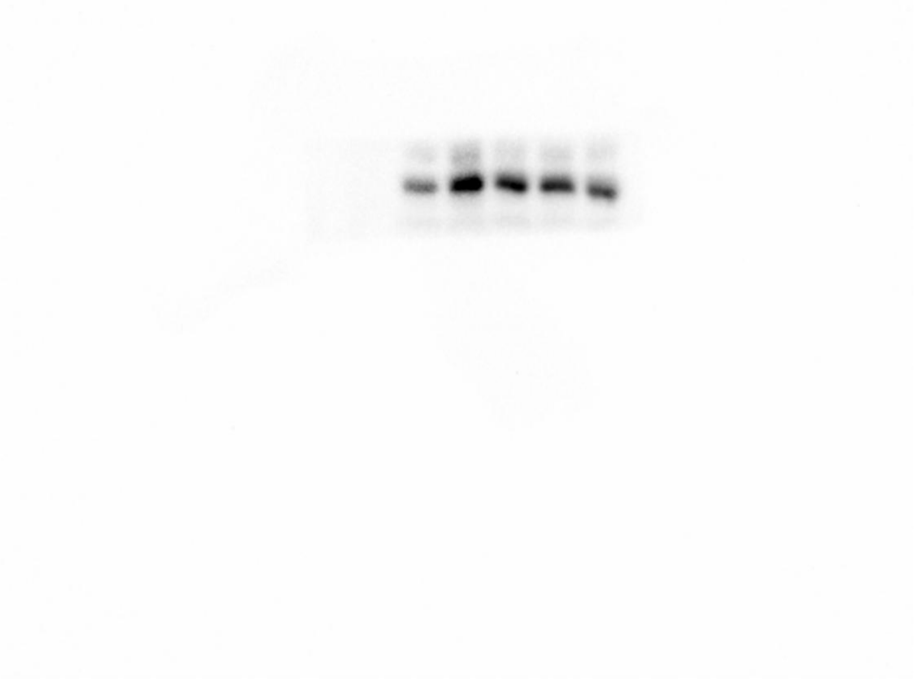

Iκbα in Figure3

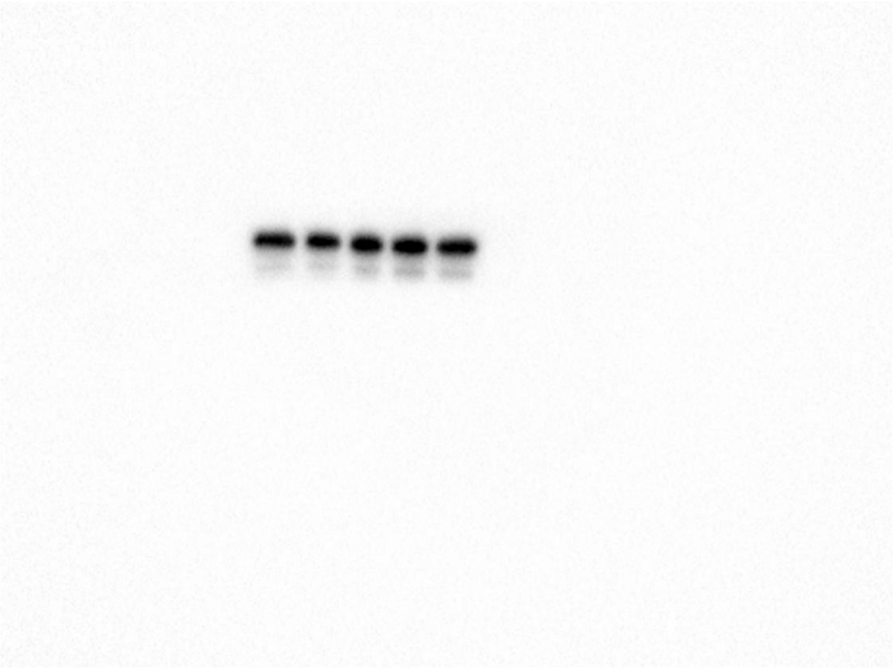

p-Iκbα in Figure3

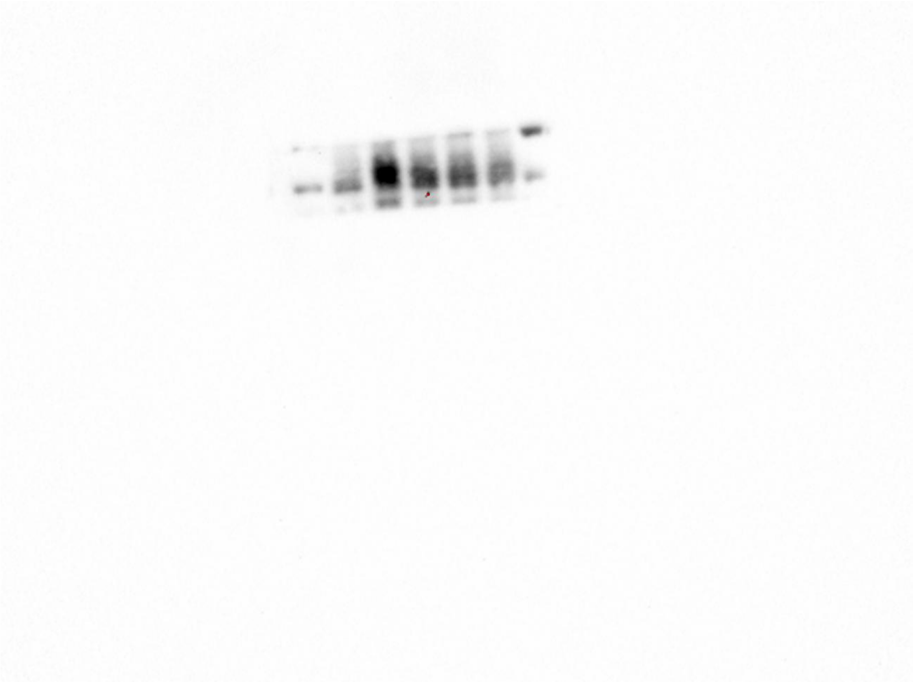

p65 in Figure3

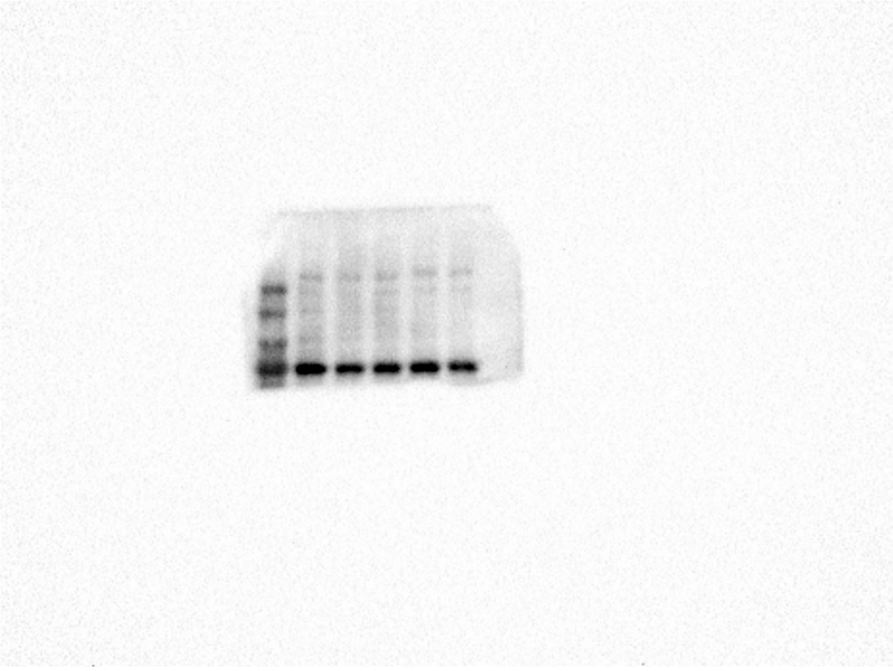

p-p65 in Figure3

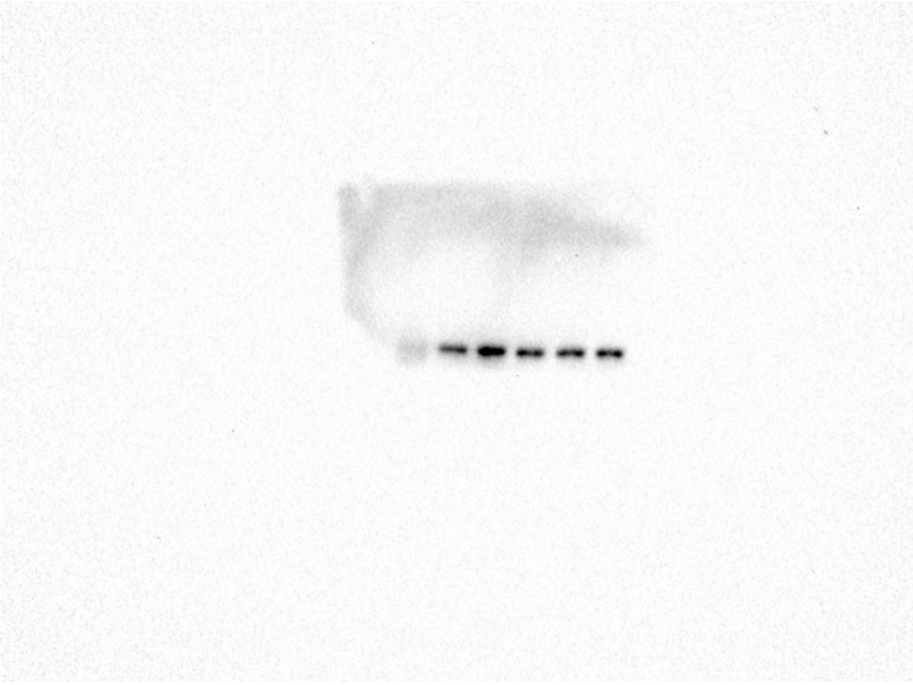

GAPDH in Figure4

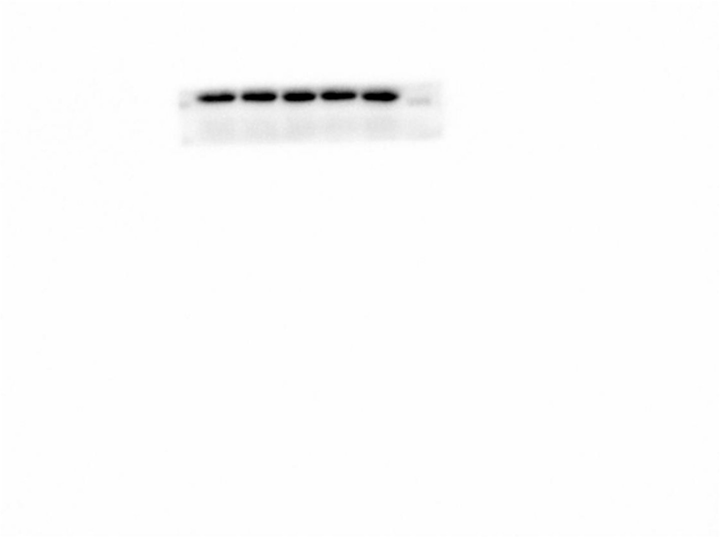

p-Iκbα in Figure4

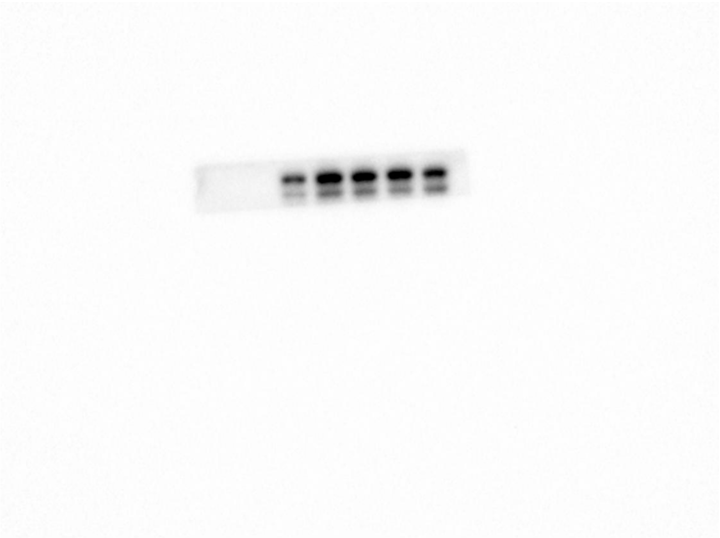

Iκbα in Figure4

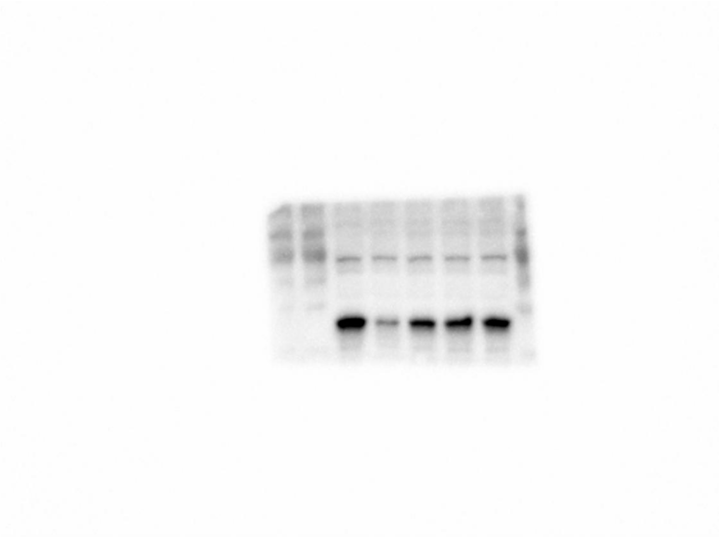

p65 in Figure4

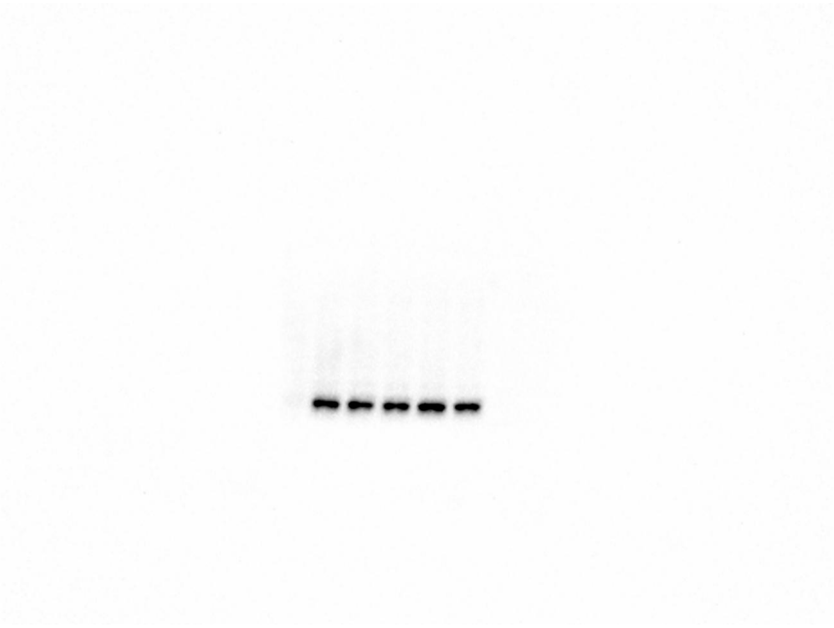

p-p65 in Figure4

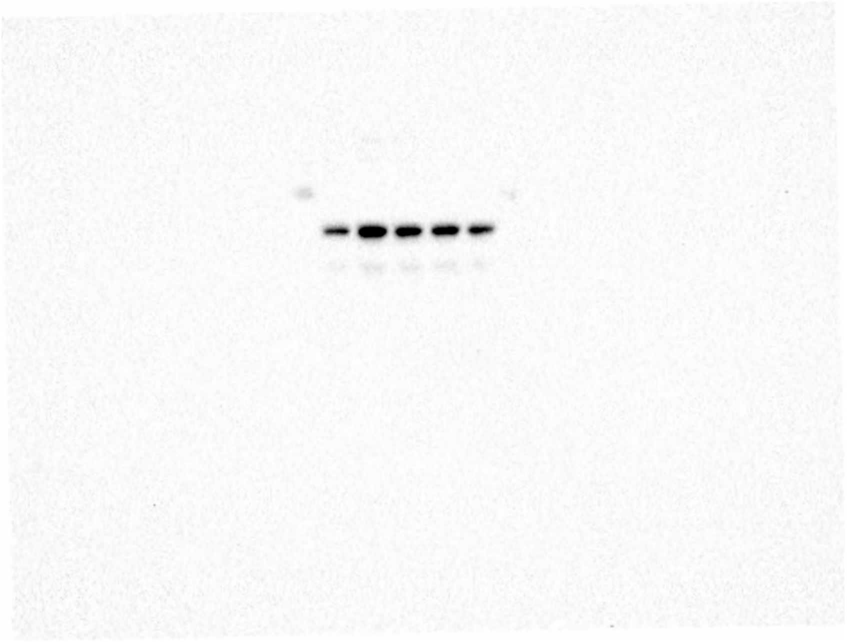

GAPDH in Figure5

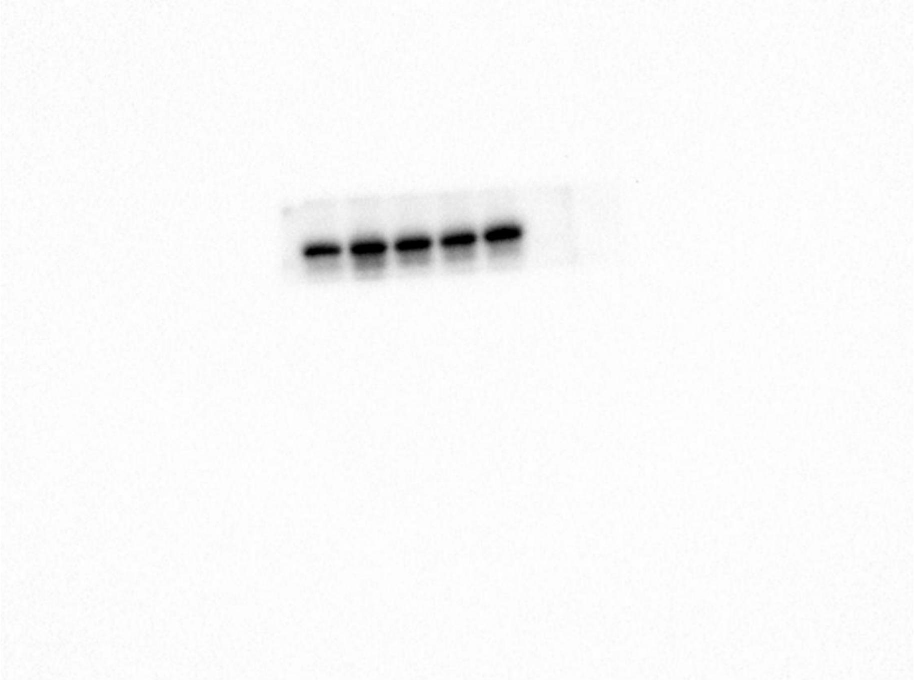

RIP1 in Figure5

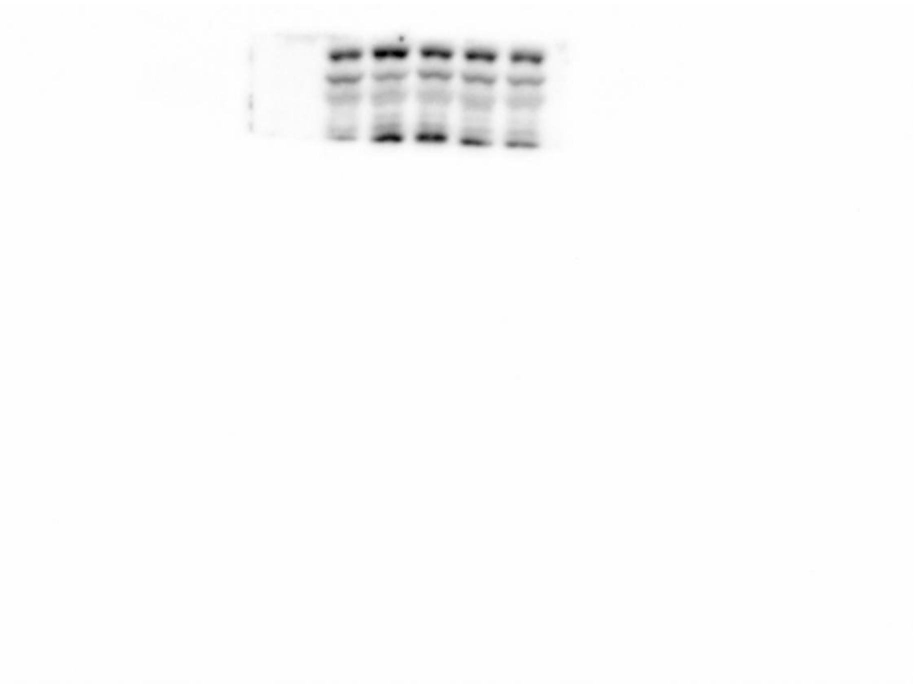

RIP3 in Figure5

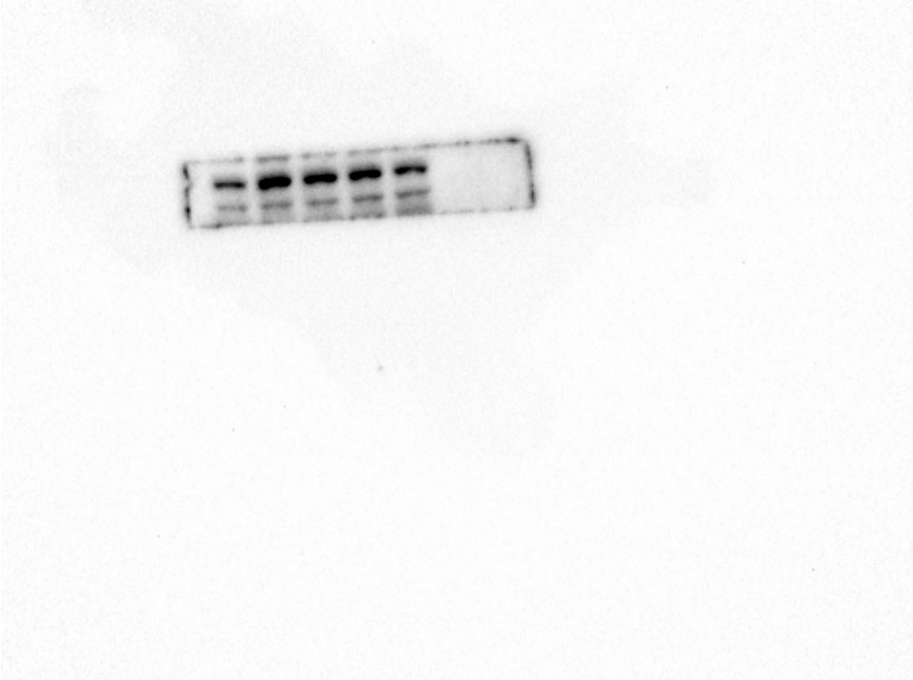

MLKL in Figure5

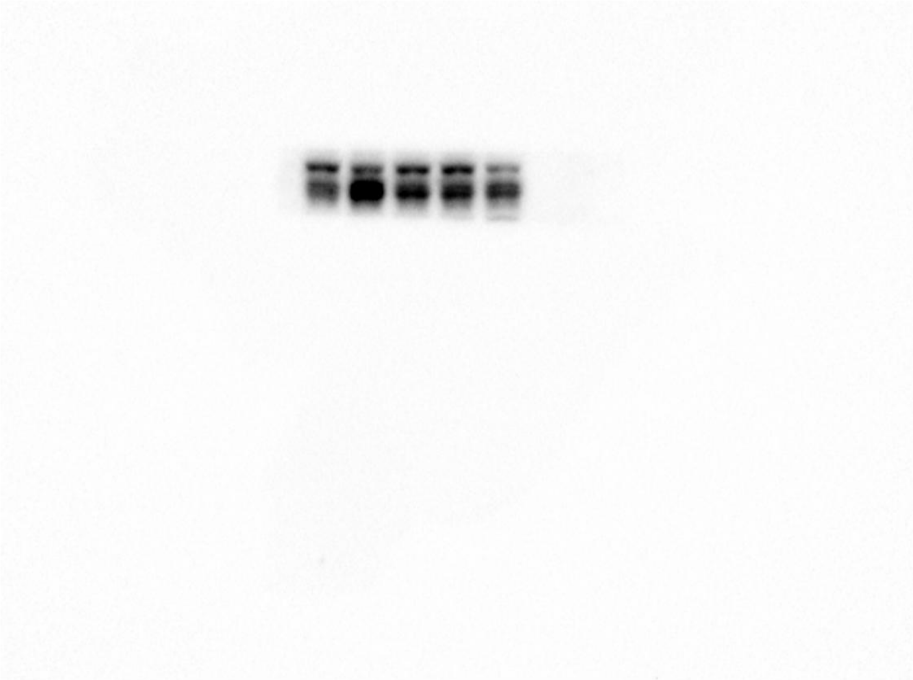

GAPDH in Figure5

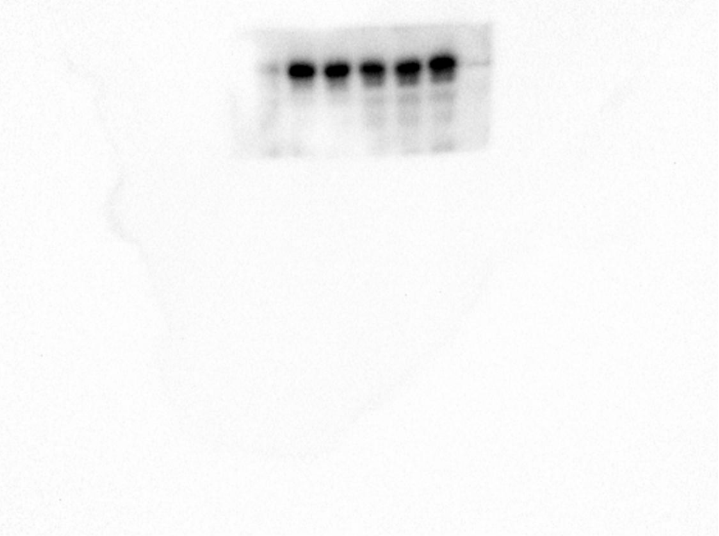

p-RIP1 in Figure5

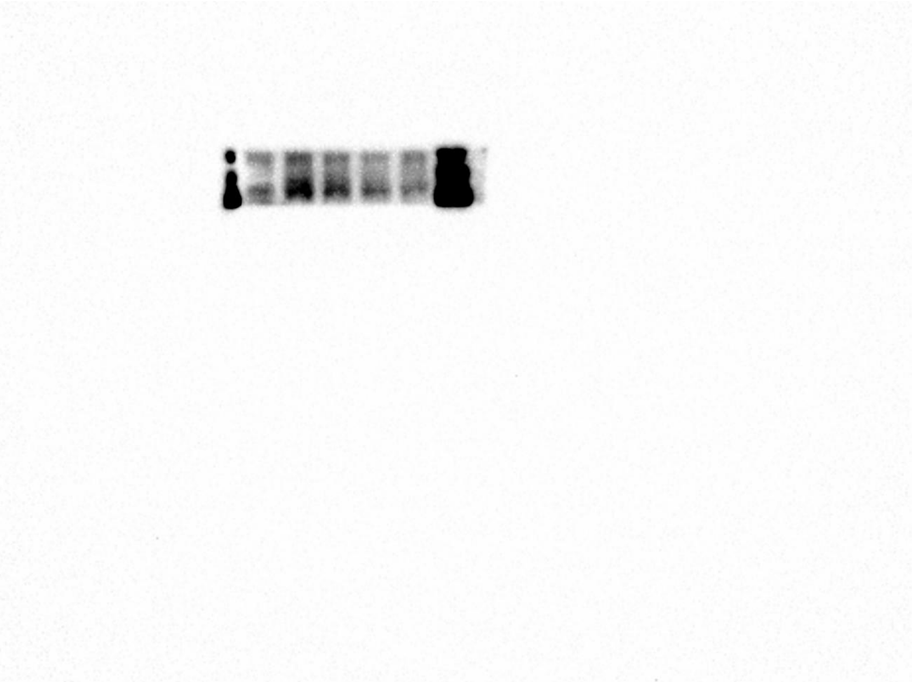

p-RIP3 in Figure5

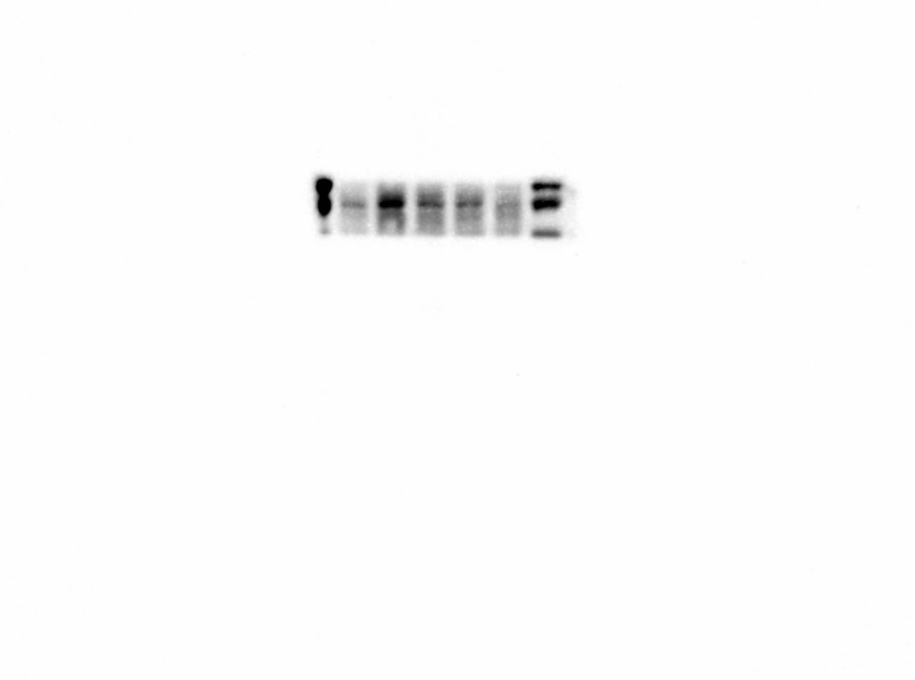

p-MLKL in Figure5

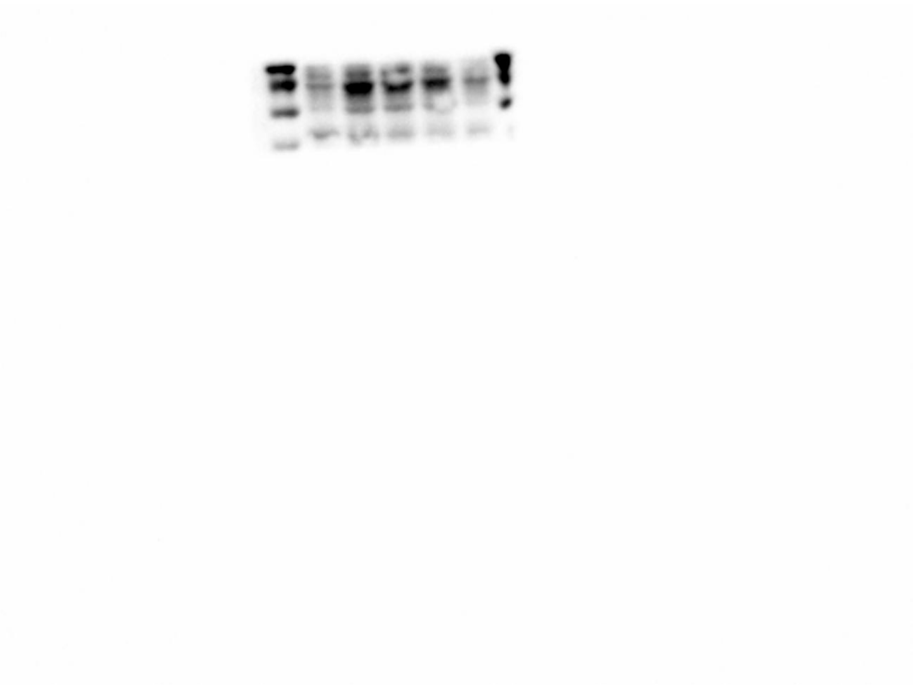

GAPDH in Figure7

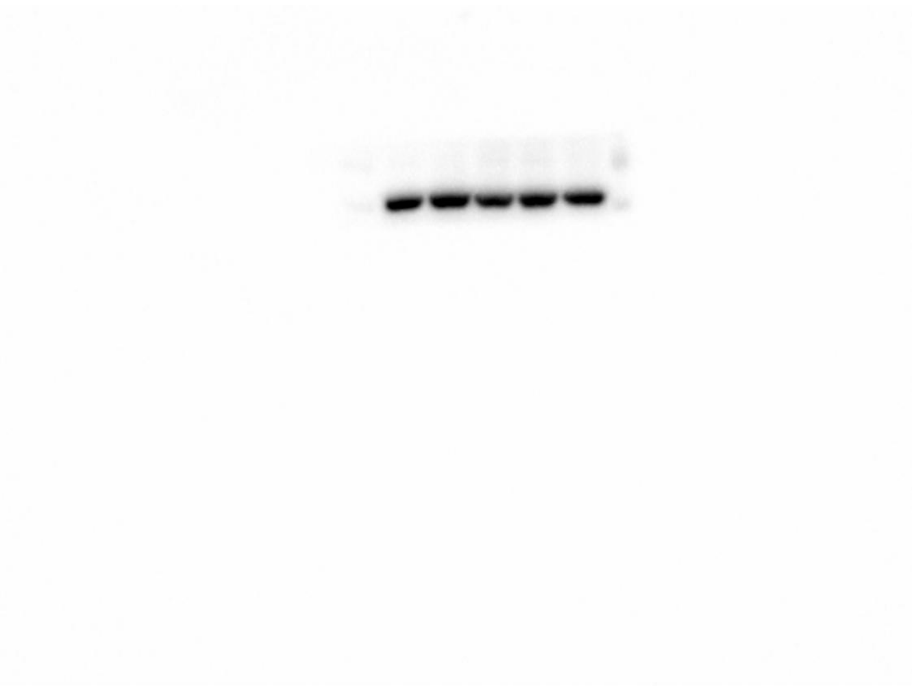

DRP1 in Figure7

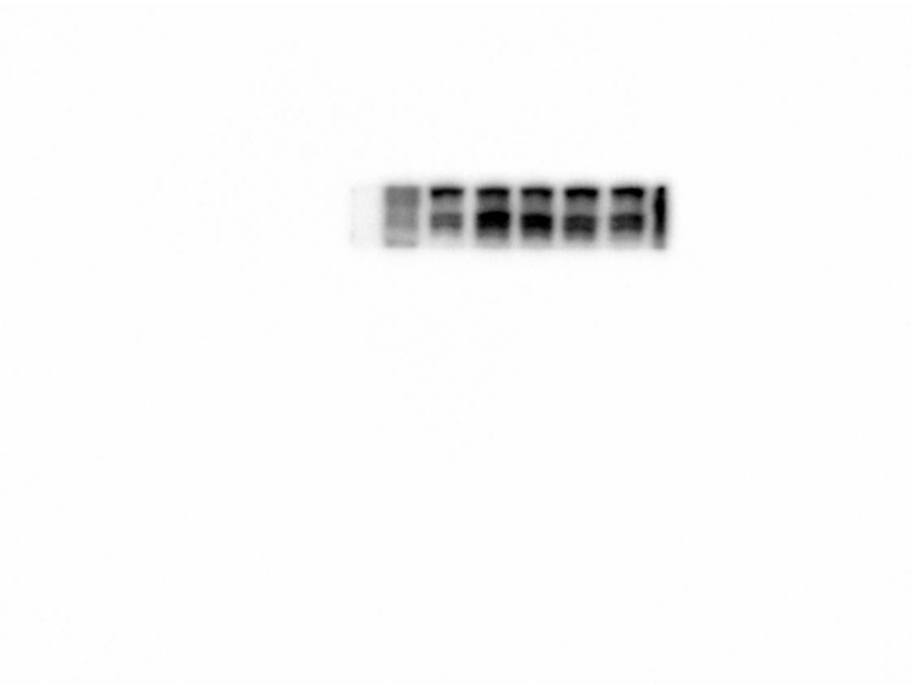

PGAM5 in Figure7

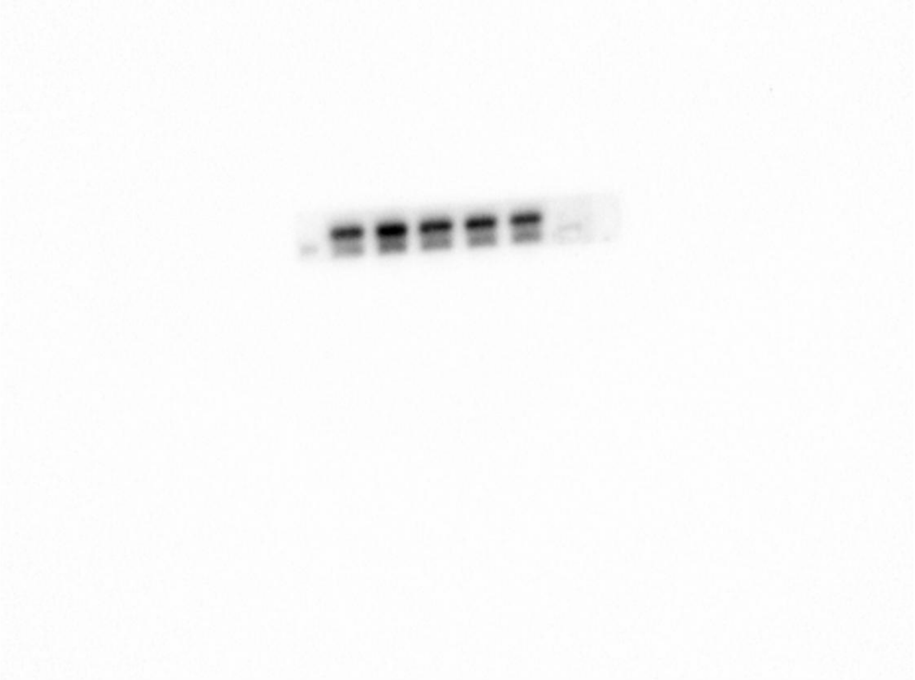

GAPDH in Figure7

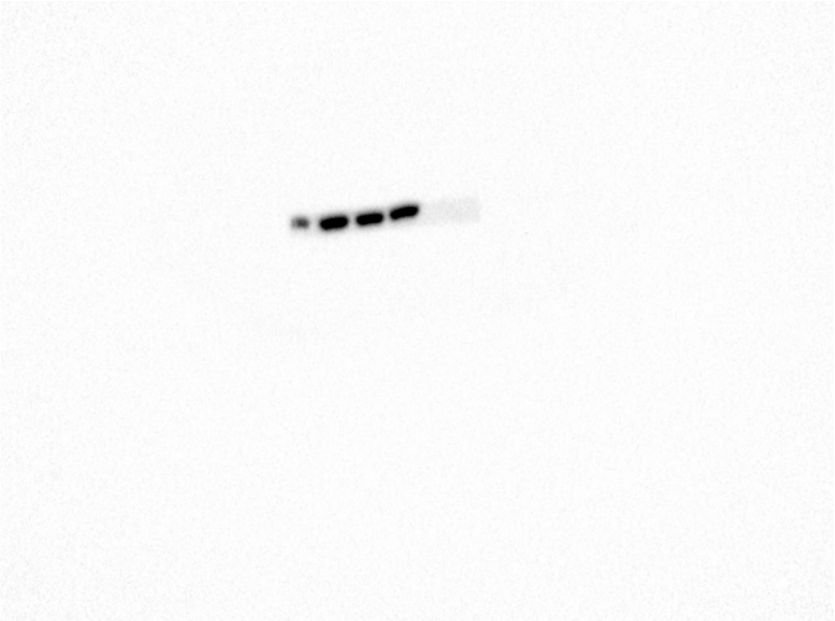

DRP1 in Figure7

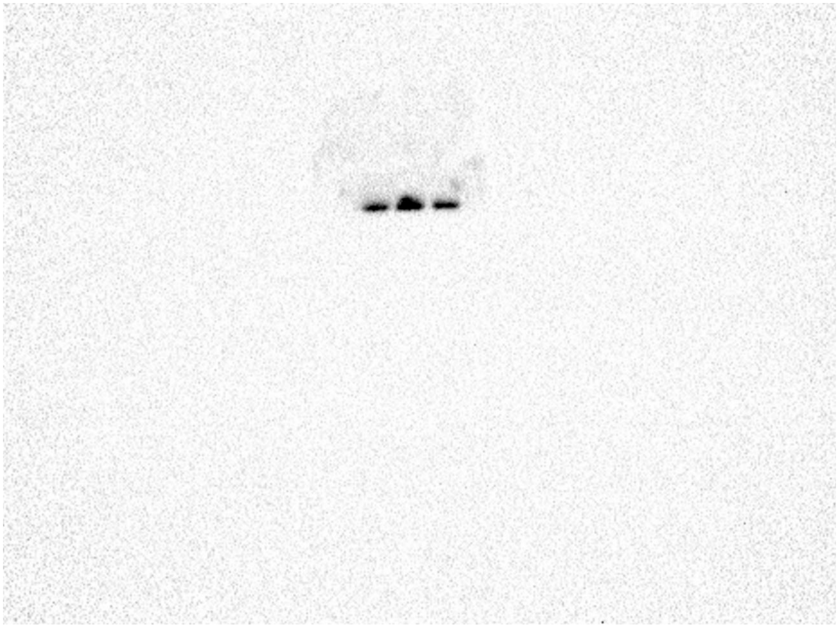

PGAM5 in Figure7

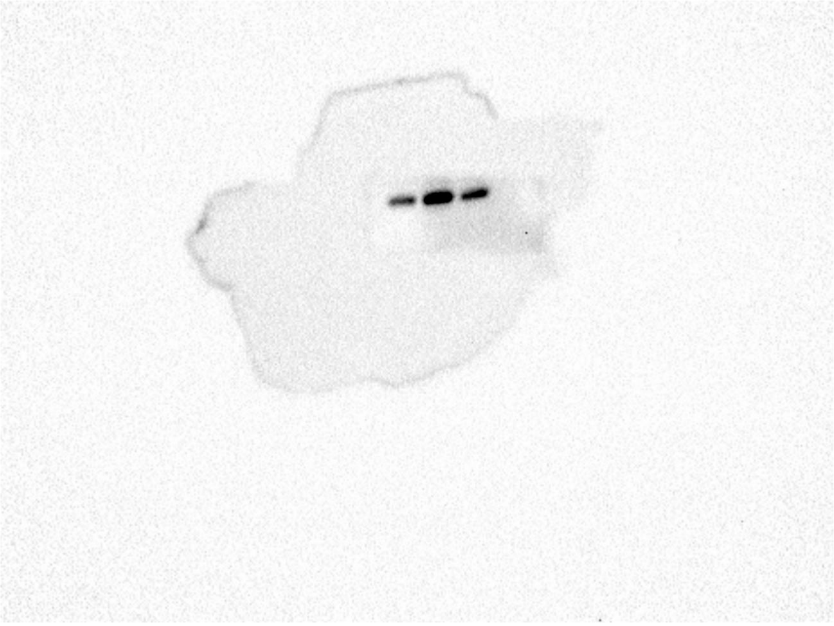

Supplement: Supplementary file 4 [file DataSheet6.PDF]
