# Supplementary material for: Acteoside attenuates RSV-induced lung injury by suppressing necroptosis and regulating metabolism
Source: Front Pharmacol. 2022 Aug 19;13:870928. doi: 10.3389/fphar.2022.870928 (PMC9437591; doi:10.3389/fphar.2022.870928)

Immunofluorescence original pictures for p-p65 in lung tissue

Control

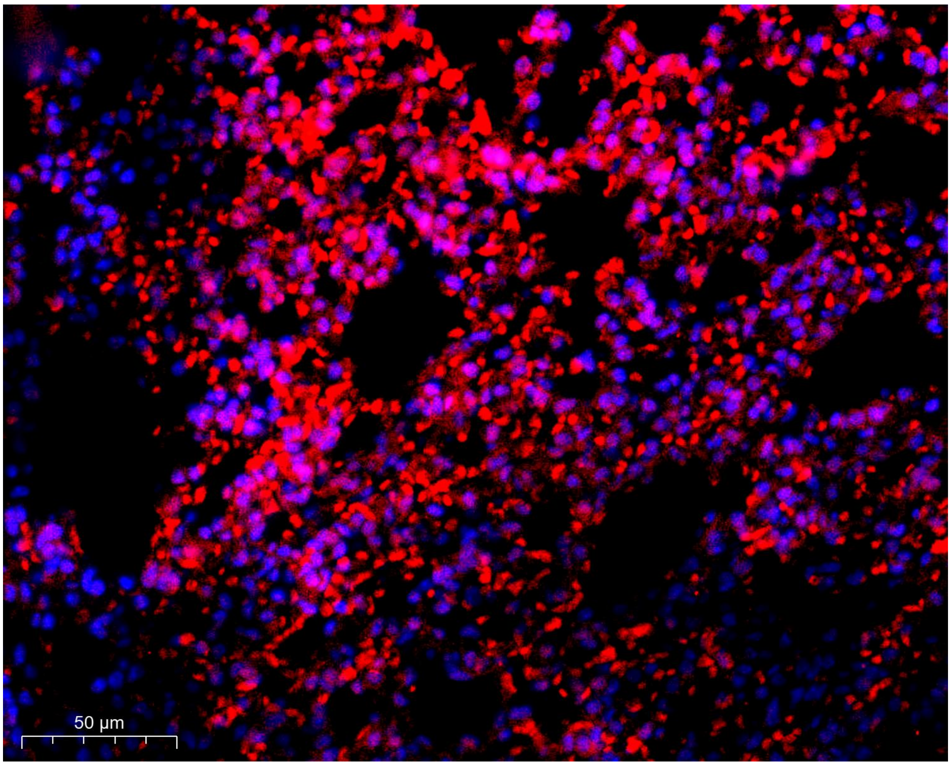

RSV

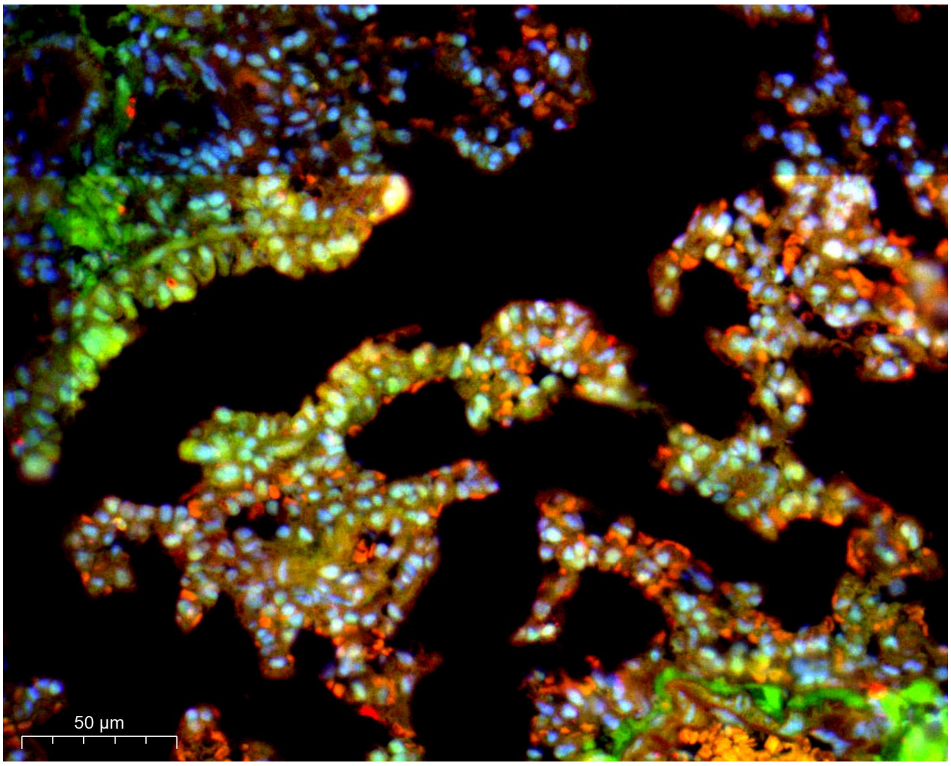

AC(90mg/kg)

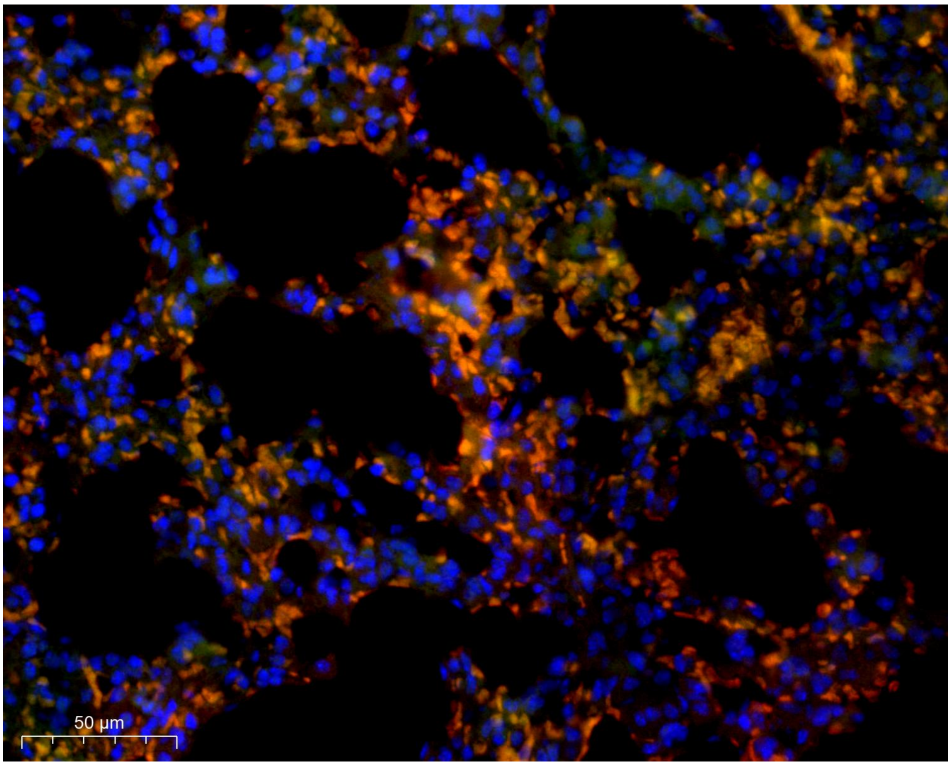

Immunofluorescence original pictures for p-MLKL in lung tissue

Control

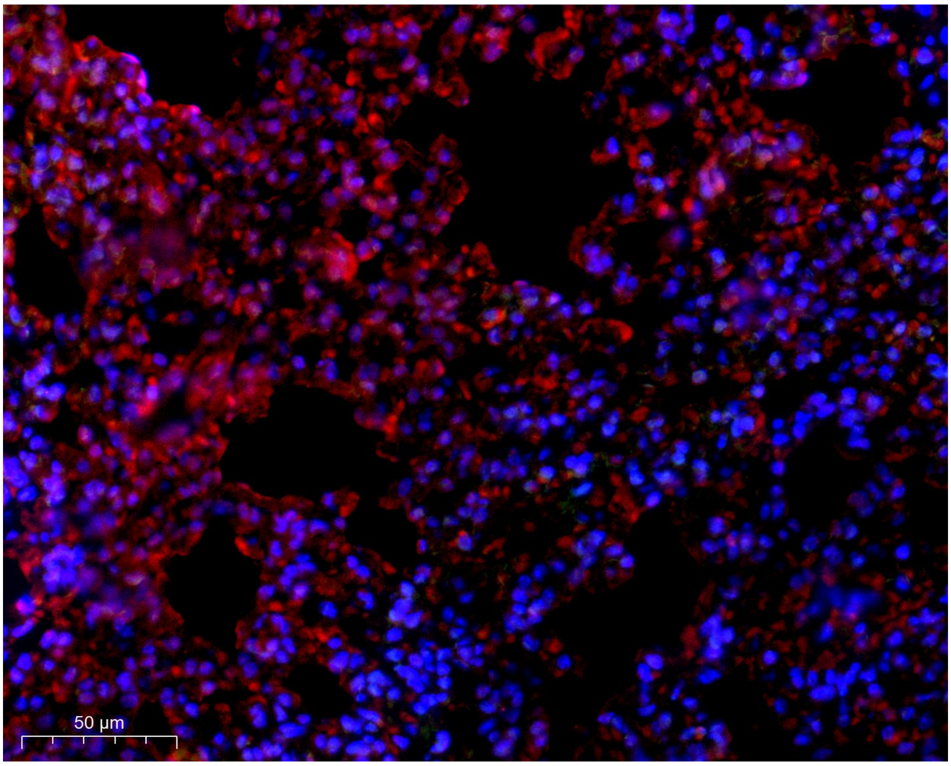

RSV

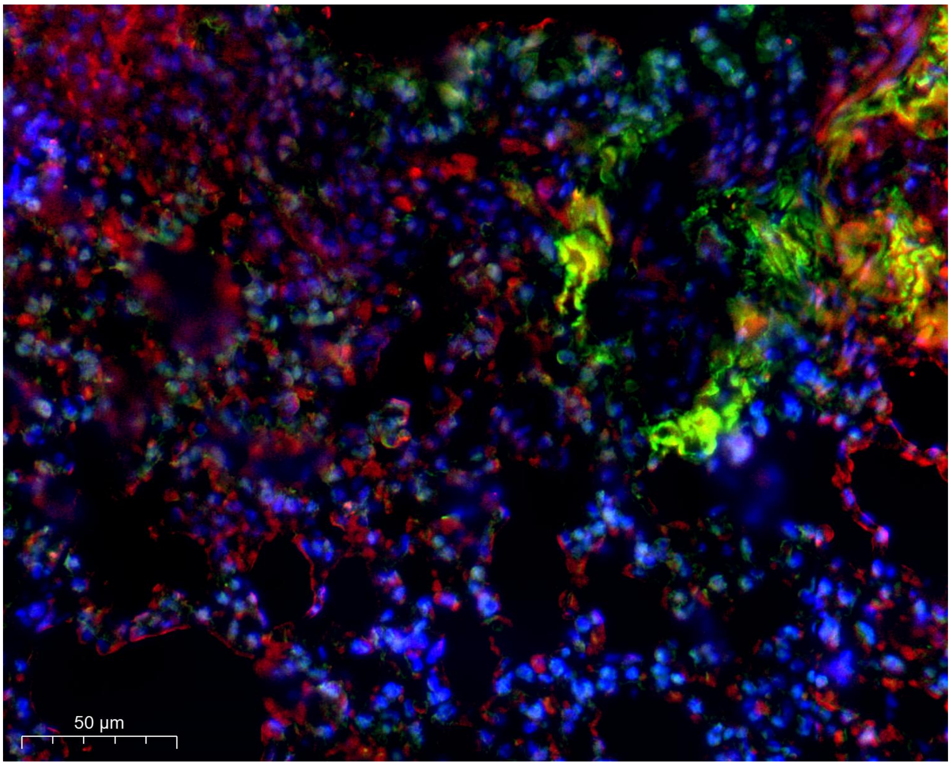

AC(90mg/kg)

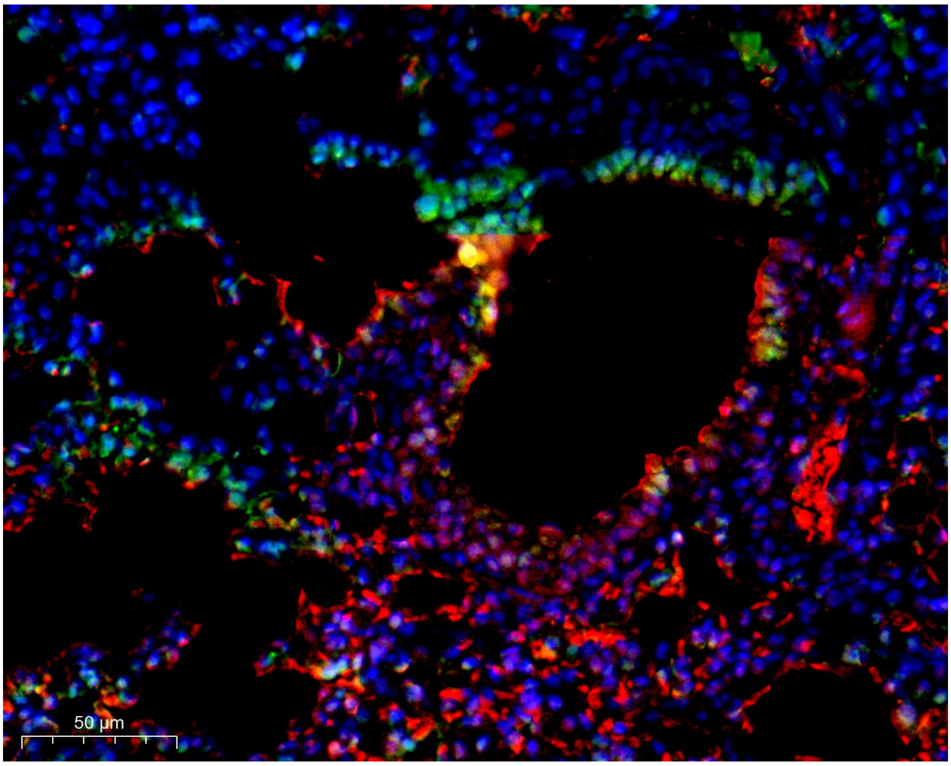

Immunofluorescence original pictures for p-MLKL in A549 cells

Control

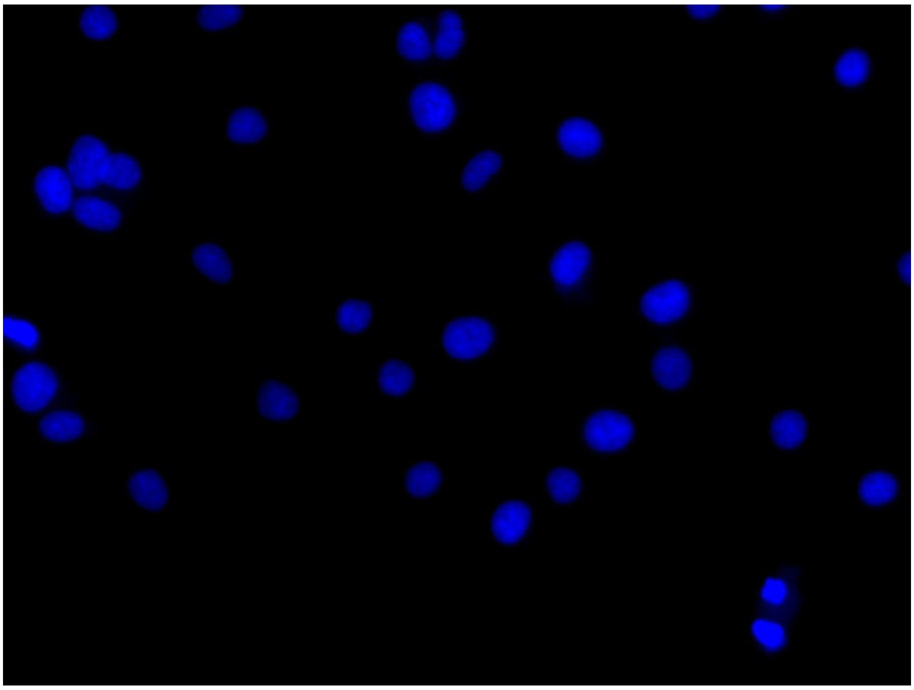

RSV

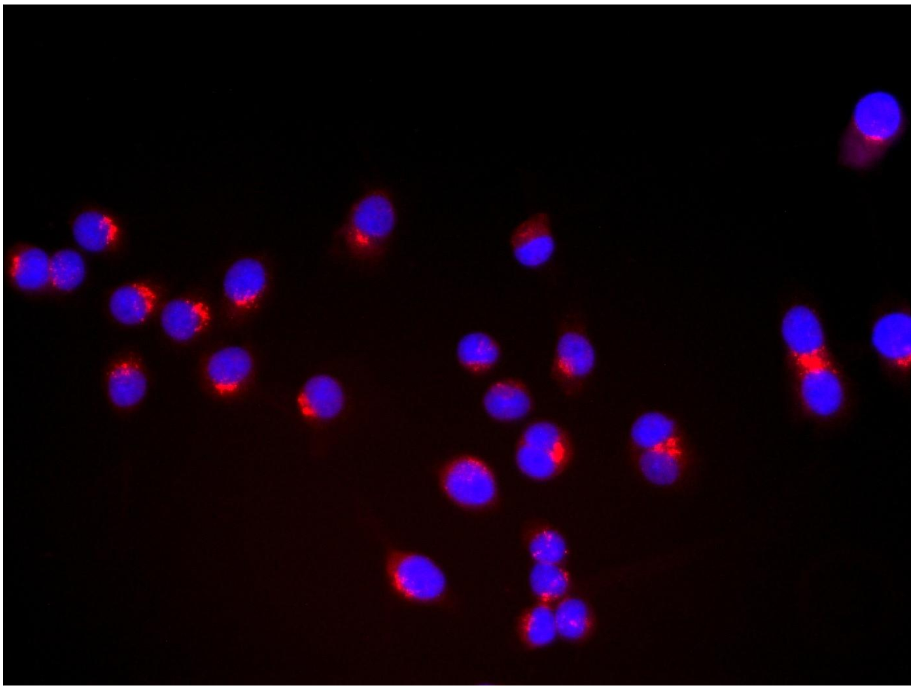

AC(5 μM)

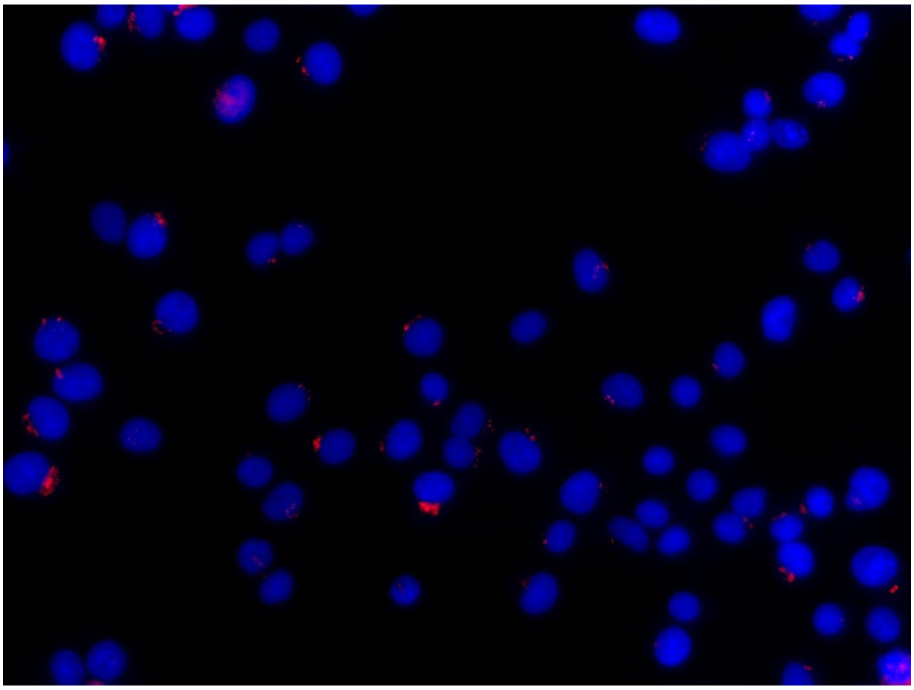

Supplement: Supplementary file 8 [file DataSheet5.PDF]
